# Histone deacetylase inhibition modulates histone acetylation at gene promoter regions and affects genome-wide gene transcription in *Schistosoma mansoni*

**DOI:** 10.1371/journal.pntd.0005539

**Published:** 2017-04-13

**Authors:** Letícia Anderson, Monete Rajão Gomes, Lucas Ferreira daSilva, Adriana da Silva Andrade Pereira, Marina M. Mourão, Christophe Romier, Raymond Pierce, Sergio Verjovski-Almeida

**Affiliations:** 1 Departamento de Bioquímica, Instituto de Química, Universidade de São Paulo, São Paulo, Brazil; 2 Laboratório de Parasitologia, Instituto Butantan, São Paulo, Brazil; 3 Grupo de Helmintologia e Malacologia Médica, Centro de Pesquisas René Rachou, Fundação Oswaldo Cruz, Belo Horizonte, Brazil; 4 Département de Biologie Structurale Intégrative, Institut de Génétique et Biologie Moléculaire et Cellulaire (IGBMC), Université de Strasbourg, CNRS, INSERM, Illkirch, France; 5 Université de Lille, CNRS, Inserm, CHU Lille, Institut Pasteur de Lille, Centre d'Infection et d'Immunité de Lille, Lille, France; University of Cambridge, UNITED KINGDOM

## Abstract

**Background:**

Schistosomiasis is a parasitic disease infecting hundreds of millions of people worldwide. Treatment depends on a single drug, praziquantel, which kills the *Schistosoma* spp. parasite only at the adult stage. HDAC inhibitors (HDACi) such as Trichostatin A (TSA) induce parasite mortality *in vitro* (schistosomula and adult worms), however the downstream effects of histone hyperacetylation on the parasite are not known.

**Methodology/Principal findings:**

TSA treatment of adult worms *in vitro* increased histone acetylation at H3K9ac and H3K14ac, which are transcription activation marks, not affecting the unrelated transcription repression mark H3K27me3. We investigated the effect of TSA HDACi on schistosomula gene expression at three different time points, finding a marked genome-wide change in the transcriptome profile. Gene transcription activity was correlated with changes on the chromatin acetylation mark at gene promoter regions. Moreover, combining expression data with ChIP-Seq public data for schistosomula, we found that differentially expressed genes having the H3K4me3 mark at their promoter region in general showed transcription activation upon HDACi treatment, compared with those without the mark, which showed transcription down-regulation. Affected genes are enriched for DNA replication processes, most of them being up-regulated. Twenty out of 22 genes encoding proteins involved in reducing reactive oxygen species accumulation were down-regulated. Dozens of genes encoding proteins with histone reader motifs were changed, including SmEED from the PRC2 complex. We targeted SmEZH2 methyltransferase PRC2 component with a new EZH2 inhibitor (GSK343) and showed a synergistic effect with TSA, significantly increasing schistosomula mortality.

**Conclusions/Significance:**

Genome-wide gene expression analyses have identified important pathways and cellular functions that were affected and may explain the schistosomicidal effect of TSA HDACi. The change in expression of dozens of histone reader genes involved in regulation of the epigenetic program in *S*. *mansoni* can be used as a starting point to look for possible novel schistosomicidal targets.

## Author summary

Human schistosomiasis is a disease caused by the parasite *Schistosoma* spp. that affects over 230 million people worldwide. Treatment depends on a single drug, praziquantel, and the search for new drugs calls for exploiting strategies that are successful for other pathologies such as cancer, including the test of inhibitors targeting chromatin enzymes responsible for modifying histone proteins associated with DNA. Histone modifications regulate cellular gene expression. Inhibitors targeting an important class of these histone-modifying enzymes, namely Histone Deacetylases (HDACs), are known to induce in vitro mortality of the parasite (at the schistosomula and adult worm stages), however the molecular changes caused in the parasite were not known. In this scenario, we studied the effect of the HDAC inhibitor Trichostatin A on the parasite genome-wide gene expression, on histone modifications at gene promoter regions and on the chromatin acetylation status, and found important affected gene pathways. In addition, this approach showed affected genes associated with other histone modifications, which led us to test and identify a synergistic schistosomicidal agent, GSK343, an EZH2 histone methyltransferase inhibitor. Our work points to the class of histone methyltransferase modifying enzyme as a novel drug target to be explored in the future for parasitosis treatment.

## Introduction

It has been widely recognized in recent years that epigenetic effectors of chromatin remodeling are promising targets for therapeutic intervention, because they play a key role in epigenetic regulation of gene expression in all eukaryotes [1,2]. For schistosomiasis, new therapeutic interventions are highly desirable [3] because it is a parasitic disease that affects over 250 million individuals worldwide [4,5], praziquantel is the only approved drug available for treatment [6] and resistant isolates of the *Schistosoma mansoni* parasite have been identified [7,8].

Chromatin is a complex structure of DNA packed into strings of nucleosomes, which are comprised of histone proteins that compact the eukaryotic genome and also regulate DNA accessibility to transcription, recombination, DNA repair and replication [9]. A range of modifications on the amino-terminal tail of histones, such as acetylation, methylation, ubiquitination, phosphorylation and sumoylation, are involved in chromatin remodeling and transcription regulation. These histone modifications are dynamically laid down and removed by histone modifying enzymes (HMEs) [10].

Two antagonistic enzyme families act to control the dynamics of histone acetylation, namely histone acetyltransferases (HATs) and histone deacetylases (HDACs) [11], thus regulating many cellular processes such as nucleosome assembly, folding of chromatin and gene transcription [12]. In the past decade, HDACs have emerged as promising targets for epigenetic-based therapies intended to reverse aberrant epigenetic states associated with cancer; similar to the large majority of anticancer drugs, HDAC inhibitors (HDACi) induce tumor cell death [13,14].

Schistosome HDACs were characterized and studied in recent years as potential new drug targets, with the strategy of testing known HDAC-inhibiting anti-cancer drugs to kill schistosomes [15–17]. The rationale of the approach is based on the fact that the parasite shares some of the characteristics of malignant cells, such as high levels of metabolic activity and of cell division, an effective host immune evasion, and an intense oxidative metabolism [18]. In fact, it is already known that all HDAC classes can be inhibited by Trichostatin A (TSA) in human cells [1], and that the parasite treatment with TSA leads to epigenetic changes in the chromatin and guides the parasite to apoptosis [15]. Also, *in vitro* assays have identified new compounds that inhibit *Sm*HDAC8 (class I) [17] and *Sm*Sirtuins (class III) [19] deacetylases. In addition, *in silico* analyses [20] have pointed to a large number of *S*. *mansoni* histone binding partners potentially involved in the regulation of gene expression, DNA replication, cell death, cellular growth and proliferation [20], thus suggesting that drug-induced histone modifications could affect these cellular processes in the parasite.

In the present study, we report the histone acetylation status and the large-scale gene expression transition promoted by TSA HDACi, and confirm the chromatin acetylation changes in some of the gene loci with altered levels of transcription. Further, our gene expression analyses have pointed to the Polycomb Repressive Complex 2 (PRC2) as being significantly affected by TSA, and this led us to test GSK343 [21], an inhibitor of EZH2, the histone methyltransferase component of PRC2, as a possible schistosomicidal compound. Indeed, we found that the GSK343 EZH2 inhibitor was active *in vitro* against *S*. *mansoni* and acted synergistically with TSA, significantly increasing parasite death.

## Methods

### Ethics statement

Animal experimentation was conducted in accordance with the Ethical Principles in Animal Research adopted by the Brazilian College of Animal Experimentation (COBEA), and the protocol/experiments have been approved by the Ethics Committee for Animal Experimentation of Instituto Butantan (CEUAIB n° 4704040515).

### Treatment of schistosomula and adult worms with HDACi

*S*. *mansoni* is maintained in the laboratory using the intermediate snail host *Biomphalaria glabrata* and as definitive host the golden hamster (*Mesocricetus auratus*). Cercariae were released from infected snails and mechanically transformed to obtain schistosomula *in vitro* [22]. Newly transformed schistosomula were maintained for 12 h in M169 (Vitrocell) medium supplemented with 2% fetal bovine serum (FBS) (Vitrocell), 1 μM serotonin, 0.5 μM hypoxanthine, 1 μM hydrocortisone, 0.2 μM triiodothyronine, penicillin/streptomycin, amphotericin, gentamicin (Vitrocell) at 37°C and 5% CO_2_ [23], after which time the drug treatment was initiated, as described below. Adult worms were obtained from 7-week infected hamsters by left ventricular perfusion, and release of worms from the hepatic portal vein. Paired worms were maintained in RPMI medium (Gibco) supplemented with 10% fetal bovine serum (FBS) (Vitrocell), penicillin/streptomycin, amphotericin (Vitrocell) at 37°C and 5% CO_2_.

The parasites were treated with 1 μM Trichostatin A (Cayman Chemical), a concentration that has been shown by Dubois et al. [15] to be sub-lethal, and the negative controls with an equivalent amount of ethanol (vehicle of TSA), for 12, 24 and 48 h for microarray experiments, and for 12 h for ChIP-qPCR and western-blotting experiments.

### Gene expression experiments

The microarray platform 4x180k was designed by our group and printed by Agilent, and it contains probes to the *S*. *mansoni* predicted genes ("Smp genes") that were annotated by the genome-sequencing project in the ASM23792v2 version of the genome [24]; the probes covered Smp genes as follows: 12 mitochondrial Smp genes, 9255 sense predicted Smp genes, in addition to probes to the opposite strand of 9079 out of the Smp 9255 predicted genes. A total of 1517 additional Smp genes predicted in the genome could not be represented by unique probes, given the recommended parameters for probe design of the Agilent microarray platform. Positive and negative control probes as well as probes for spike-in RNAs were included as recommended by the Agilent expression array design instructions (Agilent eArray). The microarray platform design along with gene annotation names was deposited at NCBI gene expression omnibus (GEO) under accession number GPL22001, and the dataset series under accession numbers GSE83208, GSE83209, GSE83210, GSE83211.

For each time point, total RNA from three biological replicates of schistosomula (treated or control) was extracted and purified using RNeasy Mini Kit (QIAGEN) according to the manufacturer’s instructions. 100 ng of each RNA sample were labeled with Cy3 or Cy5 using *Low Input Quick Amp Labeling Kit* (Agilent); the amplification method that is part of the protocol used to generate labeled cRNA is strand-specific and does preserve the strandedness of the labeled transcripts. Hybridizations were performed according to the Agilent protocols for two-color microarrays with dye-swap for technical replicates. After hybridizations and washings, microarrays were scanned with the SureScan Microarray Scanner (Agilent).

For quantitative RT-PCR, complementary DNAs were obtained by reverse transcription of 100 ng schistosomula total RNA using 6-mer random primers and SuperScriptIII Reverse Transcriptase (Invitrogen) and the qPCR amplification was done with SYBR Green Master Mix (Life Technologies) and specific primer pairs with the Applied 7500 PCR System (Applied Biosystems). Primer pairs were designed for specific *S*. *mansoni* genes by Primer3 online software (S1 Table). The results were analyzed by comparative Ct method and the statistical significance was calculated with the t-test. House-keeping gene PSMD Smp_000740 was chosen according to [25].

### Microarray data analysis

Feature Extraction Software (Agilent) was used to calculate the intensity of each spot from scanned microarray images. Raw intensity data was deposited at GEO under accession number GSE83211. The low intensity spots were filtered out from the data by applying the IsPosAndSignif flag from the Feature Extraction Software, a Boolean flag, established via a 2-sided t-test, indicating if the mean signal of a spot is greater than the corresponding background. Total intensity data were normalized by Trimmed Mean method (40%), excluding positive and negative external controls present in the platform. The log_2_ ratio between treated and control sample intensities was calculated for each spot in the array. For genes that were represented in the array by multiple probes mapping along the gene, the mean intensity signal was calculated. Pearson correlation among biological replicates and time points were calculated revealing correlation coefficients in the range 0.75 to 0.88 (Fig A in S1 Text). Significance Analysis of Microarray (SAM) [26] was used as the statistical test, applied individually for each time point using one-class approach [26]. Genes were considered as differentially expressed with q-value ≤ 0.05. Gene Ontology terms for *S*. *mansoni* genes were downloaded from the Metazoa Mart database (http://metazoa.ensembl.org/biomart/martview/) with a total of 6165 genes, and GO enrichment was calculated using Ontologizer tool [27] applying Parent-Child test with the Benjamini-Hochberg correction method [28]. To identify enriched gene networks among differentially expressed genes, QIAGEN’s Ingenuity Pathway Analysis software (IPA, QIAGEN Redwood City, www.qiagen.com/ingenuity) was used, considering 4758 *S*. *mansoni* genes encoding putative homologs to human proteins, as determined with BLASTP [29] (coverage > 20% and amino acids similarity > 40%) (S2 Table).

### Association between gene expression fold-changes and the presence of H3K4me3 mark at the promoter of genes

We assessed the H3K4me3 ChIP-Seq data by Roquis et al. [30] that was generated from schistosomula obtained 21 h after transformation of cercariae; we downloaded their raw data SRX1113460, mapped them to the *S*. *mansoni* genome version ASM23792v2 using the HOMER pipeline [31], which employs Bowtie2 to perform reads mapping and calculates significantly enriched peaks by requiring that each significant peak read density should be at least 4-fold higher than the peaks density in the surrounding 10 kb region [31]. The genomic coordinates of significant H3K4me3 peaks were associated with the genomic coordinates of the transcription start site (TSS) for known Smp genes using BedTools [32] within a window of ± 500 bp, as we have previously described [33]; in this way we were able to associate the presence of significant H3K4me3 marks to 4525 Smp genes in their promoter regions (± 500 bp of Smp gene 5´-end) (S2 Table). We tested whether the genes showing differential expression in the presence of TSA and having the H3K4me3 transcription start site mark at their TSS region had a higher mean fold-change in expression compared with the genes without the presence of this mark. For this purpose, we compared the mean fold-change (treated/control) between the two groups, namely differentially expressed genes that had a significantly enriched H3K4me3 mark at their TSS and differentially expressed genes that had no H3K4me3 mark, and applied the statistical t-test (*p*-value < 0.05).

### Protein readers of histone post-translational modifications

We used reader histone motifs from the Conserved Domains Database (CDD) (https://www.ncbi.nlm.nih.gov/cdd) to identify all *S*. *mansoni* proteins that would be predicted to recognize lysine and arginine modified by methylation and acetylation, and serine modified by phosphorylation. For this approach, we used Blastp (https://www.ncbi.nlm.nih.gov/blast) with parasite proteins as query and CDD files as subject, applying a 1e-10 cutoff of significance of alignment. S2 Table exhibits Smps with histone reader motif found in this analysis.

### Western blotting

Adult worms (treated or control) were used to prepare histone acid extracts. 50 worm pairs were soft lysed with 500 μl lysis buffer (PBS containing 0.5% Triton X-10, 0.02% NaN_3_ and Mini Protease Inhibitor Cocktail—Complete from Roche) in a glass Potter homogenizer. The samples were centrifuged (10 min, 2000 g at 4°C) and pellets containing the nuclear material were washed once in 200 μl lysis buffer then centrifuged again [15]. Histones were extracted from the nuclear fraction by suspending the pellet in 400 μl 0.25 M HCl with protease inhibitor and the solution was incubated overnight at 4°C in order to precipitate acid proteins [34]. The samples were centrifuged (60 min, 16000 g at 4°C) and the supernatants (with histone proteins) were concentrated with trichloroacetic acid 33% [35]. The final pellet with histones was eluted in MilliQ water with protease inhibitors and protein concentration was determined with the Micro BCA Protein Assay kit (Pierce Biotechnology). Of each sample, 10 μg of histone enriched extract was loaded on 15% SDS-Polyacrylamide gels, and after protein separation, transferred to a nitrocellulose membrane (Amersham). Briefly, membranes were blocked with Tris-buffered saline (TBS) containing 0.1% Tween 20 and 5% skimmed milk (TBST/5% milk), and then probed overnight with primary antibodies in TBS/2% BSA. Membranes were washed with TBST and incubated for 1 h in TBST/5% milk with secondary antibody conjugated with IRDye (IRDye 800CW goat anti-rabbit and IRDye 700CW goat anti-mouse from Licor Biosciences). After washing the membranes in TBST, the bands were visualized and quantified with the Odyssey Infrared Imaging System (Licor Biosciences). Acetylation of histones was measured with specific monoclonal antibodies to the following lysine modifications: Histone H3 acetyl K9 C5B11 (Cell Signaling) (1:1000), Histone H3 acetyl K14 ab52946 (Abcam) (1:1000), Histone H3 tri methyl K27 ab6002 (Abcam) (1:1000) and to normalize the samples anti-Histone H3 ab24834 (Abcam) (1:1000) was used.

### Chromatin immunoprecipitation coupled to detection by qPCR

The ChIP protocol for crosslinking and sonication of schistosomula was based on a protocol described elsewhere [36]. The parasite suspension was sonicated using Epishear (Active Motif) with a 3 mm microprobe with 20% amplitude, 10 pulses of 30 s each, shearing the DNA into 100–1000 bp. The immunoprecipitation was performed with EZ-Magna ChIP Chromatin Immunoprecipitation kit (Millipore) with the following antibodies: Anti-Histone H3 (Abcam), Histone H3 acetyl K9 C5B11 (Cell Signaling), Histone H3 acetyl K14 ab52946 (Abcam), Histone H3 tri methyl K27 ab6002 (Abcam), Normal mouse IgG 12-371B (Millipore) and Normal Rabbit IgG PP64B (Millipore). The recovered DNA in the precipitates was detected by qPCR with SYBR Advantage qPCR Premix (Clontech) and primers designed to specific gene promoter regions of interest (S1 Table).

We targeted these primers to approximately 500 bp upstream of the coding sequence, based on the fact that the H3K4me3 ChIP-Seq data for schistosomula from Roquis et al. [30], when mapped to the *S*. *mansoni* genome as previously described [33], falls within 500 bp of the transcription start site (TSS) of transcripts detected by RNA-Seq [33].

Primers were designed to non-repetitive regions within the promoter region of the selected set of genes indicated in the figure, with only one exception, Smp_174840 (*Sm*CBX5), a gene for which the genomic upstream TSS region is highly repetitive; in this case, we designed primers at the first exon of the *Sm*CBX5 gene. As a qPCR normalizer control we used the gene promoter region for the *Sm*Val19 gene (Smp_123090), which was not expressed either in the HDACi- or the vehicle-treated schistosomula assays, and has no histone acetylation and methylation marks at its promoter region, as seen in the public ChIP-Seq datasets from [37] available at the *Schistosoma* genome browser (http://schistosoma.usp.br).

### Parasite viability

Schistosomula were equally distributed in 96-well microtiter plates (300 larvae per well), and the drugs (TSA, GSK343 or a combination of the two) or the corresponding vehicle (control) were added, as indicated in the legends to the figures. At each time point indicated in the figures, the parasites (from a given set of wells in the plate) were stained with 2 μg/mL propidium iodide (PI) and visualized at 10 x magnification using a Nikon Eclipse fluorescent inverted microscope. Dead parasites become stained with PI and were detected with a rhodamine filter (536 nm), and total parasites inside the well were counted using light optical microscopy [38]. For each time point a new set of wells was used, because the staining procedure was lethal to the parasites. The number of biological replicates that were assayed, as well as the number of parasites that were counted per replicate, is stated in the legends to the figures. For the LD50 assay, incubation with GSK343 was maintained for 96 h before counting. For the assay of synergy between TSA and GSK343, parasites viability was measured each day along 4 days. Data were analyzed with *Origin* software (OriginLab, Northampton, MA).

### Homology modeling of SmEZH2 and molecular docking of SAM cofactor and inhibitors

The amino acid sequence of SmEZH2 (Smp_078900) was used for the identification of template structures of SET domain using Blast algorithm at RCSB Protein Data Bank (PDB) [39]. Two PDB structures of human EZH2 SET domain, 4MI0 and 4MI5, showed 63.8% and 64.9% amino acid sequence identity and 90.9% and 91.8% coverage, respectively, when compared with SmEZH2 SET domain (from amino acids 746 to 978) using the EMBOSS Needle tool (http://www.ebi.ac.uk/Tools/psa/emboss_needle/). UCSF Chimera [40] was used to generate a superimposed model from the two PDB structures with the MatchMaker tool and Needleman-Wunsch algorithm. The sequence of SmEZH2 SET domain was aligned with the model using Clustal Omega. This sequence alignment was used to obtain twenty virtual structural models with Modeller 9v10 [41], from which we selected the one with the lowest normalized DOPE-score (zDOPE, Z-score of Discrete Optimized Protein Energy). The software SCWRL4.0 [42] was applied to the selected virtual model to improve protein side-chain conformations and KobaMIM [43] was used to refine the structure. Finally the virtual model of SmEZH2 SET domain was analyzed with Molprobity [44] and ERRAT [45]. To perform molecular docking we used the previous knowledge of amino acids of hEZH2 that interact with SAM cofactor [46] to set a grid box of 30x30x30 Å around this region, in the virtual model of SmEHZ2, using the AutoDock Vina software [47]. We used the 3D ligand structures of GSK343 (CID: 71268957), GSK926 (CID: 67466175) and SAM cofactor (CID: 34756) from PubChem (https://pubchem.ncbi.nlm.nih.gov) to simulate the protein-ligand complex and obtain binding energies. This process consisted of 10 docking simulations using the following parameters: number of binding modes equal to 20 (to maximize binding free energy calculations), search exhaustiveness of 50 and 3 kcal/mol of maximum energy difference, also receptors were considered as rigid and ligands as flexible. Binding energies are shown as mean ± S.D. calculated from the 10 docking simulations. Pymol (PyMOL Molecular Graphics System, Version 1.8 Schrödinger, LLC) was used for visualization of the three dimensional virtual model of SmEZH2 SET domain. Visualization of the two-dimensional diagram summarizing the molecular interactions between ligands and EZH2 was prepared using LigPlot program [48].

## Results

### Global histone acetylation increase induced by HDAC inhibitor in S. mansoni adult worms

**Fig 1:**
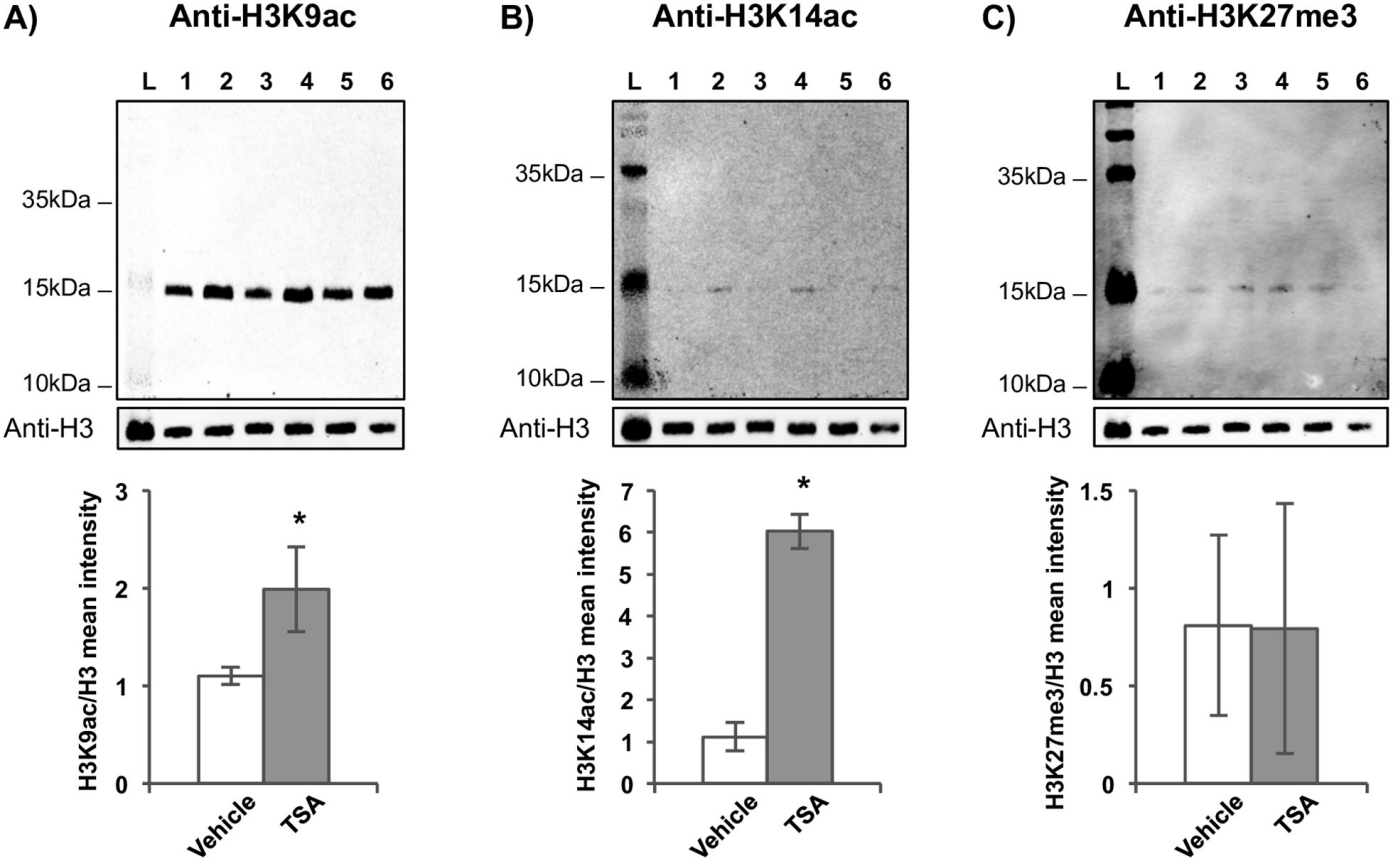
Histone H3 post-translational modifications profile in *S*. *mansoni* under HDAC inhibitor effect. Western blotting of the enriched histone protein fractions extracted from *S*. *mansoni* adult worms is shown for three biological replicates. Each biological replicate consisted of worms treated for 24 h with 1 μM TSA (lanes 2, 4 and 6) or with vehicle (lanes 1, 3 and 5). Blots were developed with (A) anti-H3K9ac, (B) anti-H3K14ac, and (C) anti-H3K27me3 antibodies. Additionally, antibody anti-H3 was used as a sample loading normalizer. Molecular weight marker ladder (L) is indicated. The graph at the lower part of each panel shows the mean intensity of the bands for the three biological replicates, obtained by extracting the intensity values of scanned images; for each sample, the intensity of the modified histone band was normalized by the intensity of histone H3. Mean ± SD is shown; t-test was applied and statistically significant *p*-values ≤ 0.05 are indicated with an asterisk.

The extent of histone acetylation in *S*. *mansoni* adult worms under the effect of the HDACi TSA was investigated after 24 h of parasite exposure to the drug. Histone marks H3K9ac and H3K14ac, associated with transcriptional activation, were studied by western blotting with monoclonal antibodies against the specific acetylated lysine 9 (K9) and lysine 14 (K14) residues of histone H3. Histone hyperacetylation was detected both at H3K9ac and H3K14ac (Fig 1A and 1B); three independent biological replicates showed a statistically significant (*p*-value ≤ 0.05) increase in acetylation. In parallel, the H3K27me3 histone mark, a non-related mark of transcription repression, was assayed as a control and found not to be affected by the TSA treatment (Fig 1C); this also suggests that no overall changes in histone modification had been triggered as a consequence of histone hyperacetylation.

### HDAC inhibition causes global gene expression changes in schistosomula

To explore the effect of HDACi on gene expression, three independent biological replicates of schistosomula were exposed *in vitro* to TSA or drug vehicle and large-scale gene expression changes were accessed by microarrays. Three different time points after drug exposure were analyzed (12, 24 and 48 h). Our custom designed strand-specific Agilent microarray platform has probes for 9255 *S*. *mansoni* protein-coding gene transcripts; in addition, probes for the opposite complementary strand are present on the microarray, to detect an eventual antisense transcription for 9079 out of the 9255 gene loci.

**Fig 2:**
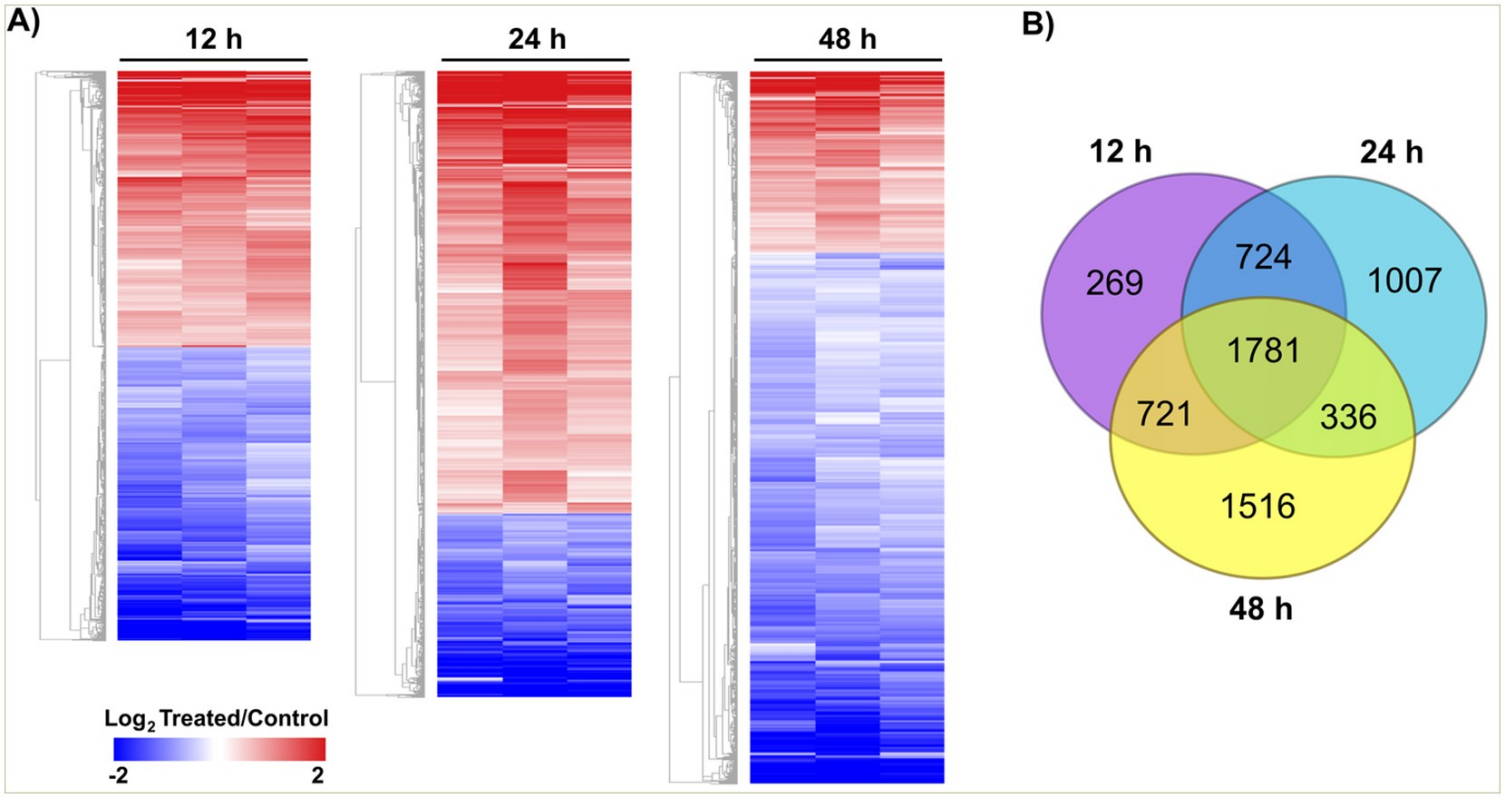
Profile of schistosomula gene expression changes at different times after exposure to HDACi. (A) Hierarchical clustering of differentially expressed genes (lines) for the three biological replicates (columns) at each of three different time points after exposure of schistosomula to the HDACi TSA (12, 24 and 48 h). The dimensions of the y-axis at each time point is proportional to the total number of differentially expressed genes at that time point. Gene expression levels are calculated as Log_2_ ratio (treated/control) and color-coded as indicated on the scale at the bottom left. (B) Venn diagram of the number of differentially expressed genes observed at each of the three time points; a total of 1781 genes were affected in common at the three time points.

The number of genes affected by exposure to the HDACi, compared with vehicle at each time point, is shown in Table 1. Note that the fraction of affected genes increased along the time of drug exposure and reached 54% within 48 h of treatment (Fig 2A, Table 1). It is interesting to note that at 24 h there was a predominant up-regulation of 2719 genes in the presence of the drug compared to vehicle, and only 1129 genes were down-regulated (Table 1, Fig 2A), while at 48 h of treatment just one quarter of the affected genes were up-regulated, greatly increasing the fraction of down-regulated genes. Venn diagrams for the subsets of up-regulated and down-regulated genes (Fig B in S1 Text) show that a large set of genes are affected exclusively at just one of the three time points analyzed. It is noteworthy that 1781 genes were affected in common at the three time points analyzed (Fig 2B and Fig C in S1 Text). Overall, the data indicate a modification of the parasite’s gene transcription program along the time course of drug exposure.

**Table 1:** Large-scale gene expression changes at three different time points after exposure of *S*. *mansoni* schistosomula to the HDAC inhibitor TSA.

			Duration of treatment		
			12 h	24 h	48 h
Expressed Smp genes (in control or treated—or in both conditions)			8198	7916	8090
Differentially expressed Smp genes compared to untreated controls ^a^ (% of total expressed Smps)			3495 (43%)	3848 (49%)	4353 (54%)
	Up-regulated Smp genes		1695	2719	1108
	Down-regulated Smp genes		1800	1129	3245
		Up-regulated Smp genes with histone reader motif	36	77	20
		Down-regulated Smp genes with histone reader motif	37	12	65
Expressed antisense RNAs (asRNAs) (in control or treated—or in both conditions)			4516	3257	3490
Differentially expressed asRNAs compared to untreated controls ^a^ (% of total expressed asRNAs)			1506 (33%)	1376 (42%)	1559 (45%)
	Up-regulated asRNAs		1137	1193	963
	Down-regulated asRNAs		369	183	596

^a^Statistically significant differential expression (q-value ≤ 0.05)

The strand-specific cRNA labeling protocol that was used here allowed the detection of transcriptional activity antisense to protein-coding genes, and indeed we detected that TSA treatment did affect antisense RNA (asRNA) transcription. Similar to the mRNAs, the fraction of affected asRNAs increased along the time of drug exposure and reached 45% of all expressed asRNAs within 48 h of treatment (Table 1); the majority of them were up-regulated in the presence of TSA (Table 1). Considering the mRNA and the asRNA from the same locus, we found that at each time point there were over 700 loci where the pattern of expression change with drug was the same for both strands, namely both mRNA and asRNA were simultaneously up-regulated or both were down-regulated (Table A in S1 Text) upon TSA exposure. In addition, at each time point we detected over 400 loci where only the asRNA was differentially expressed by exposure of schistosomula to TSA (most of them up-regulated), and the sense protein-coding mRNA of the same locus was not affected by the drug treatment (Table A in S1 Text).

### Genes with H3K4me3 mark at their promoter region are most highly induced by drug treatment compared with those without the mark

**Fig 3:**
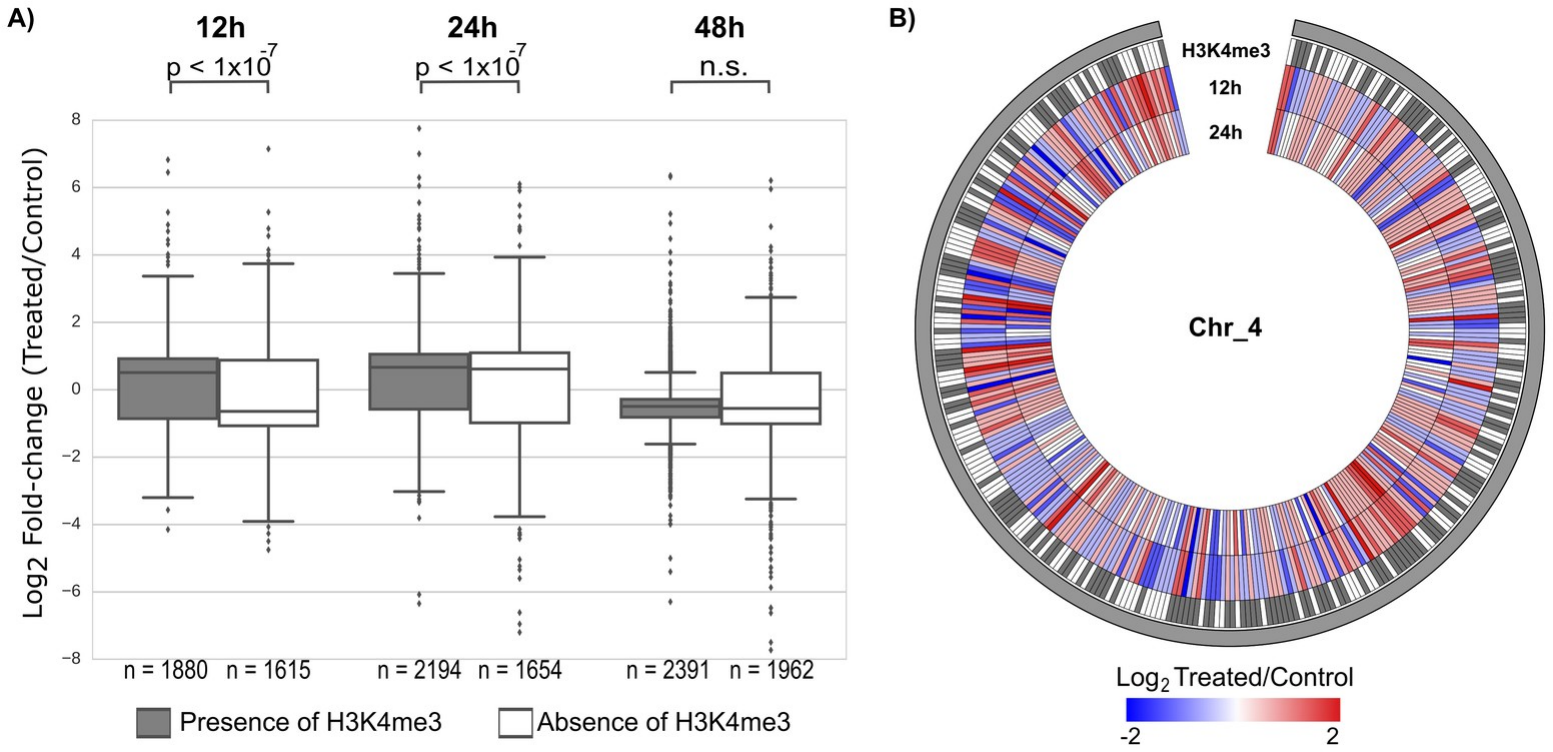
Presence of H3K4me3 at promoter region correlates with larger gene expression change upon HDACi treatment. (A) Boxplot of expression fold-change (log_2_ treated/control) for all genes that were affected by TSA treatment of schistosomula. Genes were grouped into two categories according to the presence (grey box) or absence (white box) of a significantly enriched H3K4me3 histone mark at their gene promoter region. Genes under analysis are those that were detected as differentially expressed at 12, 24 or 48 h after schistosomula TSA treatment. (B) Circos plot displaying the genomic distribution pattern of the H3K4me3 marks (grey), for all genes along chromosome 4 that were detected as significantly differentially expressed at 12 h treatment of schistosomula with TSA (q-value ≤ 0.05). Genes (12 h) were colored by the extent of expression fold-change (log_2_ treated/control), with negative values in blue and positive values in red. For comparison, the gene expression fold-changes for these genes that persisted at 24 h are also shown.

Knowing that the H3K4me3 histone mark is related to transcriptionally active chromatin, we collected the H3K4me3 ChIP-Seq data for schistosomula from Roquis et al. [30], mapped them to the *S*. *mansoni* genome as previously described [33], and asked if the genomic positions having significantly enriched H3K4me3 marks would correspond to the positions of genes that would be more susceptible to changes in expression due to HDAC inhibition by TSA. For this analysis, we categorized the differentially expressed genes according to the presence or the absence of a significantly enriched H3K4me3 mark at their promoters (see Methods), and computed the distribution of gene expression fold-change for each group at 12, 24 and 48 h after treatment of schistosomula with TSA (Fig 3A).

Interestingly, we found that at 12 h after TSA treatment (Fig 3A), genes that have the H3K4me3 histone mark at their promoters showed a median log_2_ fold-change (treated/control) of 0.55, i.e., on average they showed a median 1.5-fold activation in the presence of TSA relative to control. On the contrary, genes without the H3K4me3 mark at their promoters showed a median log_2_ fold-change of -0.61, i.e., on average they showed a median 1.5-fold inhibition in the presence of TSA relative to control (Fig 3A), a significantly different pattern from that of genes with the H3K4me3 mark (*p*-value < 1 x 10^−7^). The differences in the pattern of expression change between the two groups were still observed after 24 h of TSA (*p*-value < 1 x 10^−7^) and vanished at 48 h (Fig 3A); such a persistent difference at 24 h in the pattern of expression change between the genes with and without the H3K4me3 mark can be further appreciated with the cumulative distribution plot of Fig D in S1 Text, where the cumulative curve for genes with the mark is clearly shifted to the right, indicating a larger change in gene expression for the genes with the mark compared with the ones without. Also, the Kolmogorov-Smirnov test showed a significant difference (*p*-value ≤ 0.0001) in the distribution profiles of log_2_ fold-change (treated/control) between the two groups of genes with or without the H3K4me3 mark, at each of the two early time points.

Fig 3B shows an example of genomic distribution pattern of H3K4me3 marks along chromosome 4 for all significant differentially expressed genes at 12 h. It can be seen that a great number of differentially expressed genes are associated with the presence of the H3K4me3 mark at their promoters (Fig 3B). Fig 3B also illustrates how the pattern of activation/inhibition of the genes in chromosome 4 changed at 24 h compared with the pattern at 12 h, nevertheless most of the expression changes persisted at 24 h.

### Genes affected by HDACi are directly associated with chromatin regulation

Gene Ontology analysis pointed to distinct enriched categories for up- and down- regulated genes in schistosomula at each of the three analyzed time points after HDACi treatment (Tables 2–4). Categories associated with ATP metabolism, such as ATP catabolic process (GO:0006200), ATP binding (GO:0005524) and Purine nucleotide binding (GO: 0017076) were enriched among the up-regulated genes after 12, 24 and 48 h treatment respectively. The category of phosphorus metabolic process (GO:0006793) was enriched among the up-regulated genes at 12 and 24 h.

**Table 2:** Gene ontology categories enrichment for differentially expressed genes of schistosomula treated for 12 h with HDAC inhibitor (TSA).

Gene Ontology Category	GO ID	NSP	*p*-value^a^	Pop.	DEGs
**Genes up-regulated at 12 h treatment with TSA**					
Ciliary part	GO:0044441	C	0.0348	6	5
Phosphorus metabolic process	GO:0006793	B	0.0003	622	164
Cellular protein modification process	GO:0006464	B	0.0042	408	98
Signal transduction	GO:0007165	B	0.0076	358	89
ATP catabolic process	GO:0006200	B	0.0127	32	14
ATP binding	GO:0005524	M	0.0067	581	160
Divalent inorganic cation transmembrane transporter activity	GO:0072509	M	0.0228	18	10
Transferase activity	GO:0016740	M	0.0228	779	201
**Genes down-regulated at 12 h treatment with TSA**					
Plasma membrane	GO:0005886	C	0.0026	67	32
Cell surface receptor signaling pathway	GO:0007166	B	0.0105	122	46
Receptor activity	GO:0004872	M	0.0028	103	43
Cation binding	GO:0043169	M	0.0128	849	217

NSP, the namespace, or subontology; B, Biological process; C, Cellular component; M, Molecular function; Pop., population number of *S*. *mansoni* genes present in the GO category; DEGs, number of differentially expressed genes enriched in the category.

^a^Benjamini-Hochberg adjusted *p*-value.

**Table 3:** Gene ontology categories enrichment for differentially expressed genes of schistosomula treated for 24 h with HDAC inhibitor (TSA).

Gene Ontology Category	GO ID	NSP	*p*-value^a^	Pop.	DEGs
**Genes up-regulated at 24 h treatment with TSA**					
Transferase activity, transferring phosphorus-containing groups	GO:0016772	M	0.0152	454	220
ATP binding	GO:0005524	M	0.0435	572	267
DNA replication	GO:0006260	B	0.0154	51	33
Phosphorus metabolic process	GO:0006793	B	0.0187	610	266
**Genes down-regulated at 24 h treatment with TSA**					
Cell surface receptor signaling pathway	GO:0007166	B	0.0000	116	45
Molecular transducer activity	GO:0060089	M	0.0000	114	38
Integral component of membrane	GO:0016020	C	0.0002	537	134
Proteolysis	GO:0006508	B	0.0003	192	42
Calcium ion binding	GO:0005509	M	0.0014	177	47
Single-organism process	GO:0044699	B	0.0061	1198	216
Structural constituent of cytoskeleton	GO:0005200	M	0.0067	12	6
Signaling receptor activity	GO:0038023	M	0.0088	84	38
Transporter activity	GO:0005215	M	0.0232	266	56
Plasma membrane	GO:0005886	C	0.0324	59	18
Iron ion binding	GO:0005506	M	0.0378	22	9
O-acyltransferase activity	GO:0008374	M	0.0378	11	5
Microtubule associated complex	GO:0005875	C	0.0389	56	15

NSP, the namespace, or subontology; B, Biological process; C, Cellular component; M, Molecular function; Pop., population number of *S*. *mansoni* genes present in the GO category; DEGs, number of differentially expressed genes enriched in the category.

^a^Benjamini-Hochberg adjusted *p*-value.

**Table 4:** Gene ontology categories enrichment for differentially expressed genes of schistosomula treated for 48 h with HDAC inhibitor (TSA).

Gene Ontology Category	GO ID	NSP	*p*-value^a^	Pop.	DEGs
**Genes up-regulated at 48 h treatment with TSA**					
DNA replication	GO:0006260	B	0.0001	52	24
MCM complex	GO:0042555	C	0.0109	5	5
Purine nucleotide binding	GO:0017076	M	0.0198	715	103
Catalytic activity	GO:0003824	M	0.0371	1993	273
Cell projection part	GO:0044463	C	0.0468	5	4
**Genes down-regulated at 48 h treatment with TSA**					
Plasma membrane	GO:0005886	C	0.0000	63	49
Receptor activity	GO:0004872	M	0.0012	106	66
Calcium ion binding	GO:0005509	M	0.0065	180	101
Extracellular matrix structural constituent	GO:0005201	M	0.0134	8	8
Oxidoreductase activity	GO:0016491	M	0.0209	205	103
Cell surface receptor signaling pathway	GO:0007166	B	0.0233	126	75
GTP binding	GO:0005525	M	0.0272	135	63
Monovalent inorganic cation transport	GO:0015672	B	0.0274	75	46
Cofactor biosynthetic process	GO:0051188	B	0.0274	34	19
Cell-cell adhesion	GO:0098609	B	0.0302	44	34
Nucleosome	GO:0000786	C	0.0427	23	15

NSP, the namespace, or subontology; B, Biological process; C, Cellular component; M, Molecular function; Pop., population number of *S*. *mansoni* genes present in the GO category; DEGs, number of differentially expressed genes enriched in the category.

^a^Benjamini-Hochberg adjusted *p-value*.

Many interesting categories are involved with regulation of DNA and chromatin, such as DNA replication (GO:0006260) among the up-regulated genes at 24 and 48 h, and minichromosome maintenance (MCM) complex (GO:0042555) among the up-regulated genes after 48 h treatment. Interestingly, the nucleosome category (GO:0000786) was enriched among down-regulated genes after 48 h treatment.

Genes affected in common at the three time points of TSA treatment (1781 genes) were separated into two subsets of up-regulated or down-regulated genes (Fig C in S1 Text) and were classified into a number of enriched GO categories (Table 5), including the GO associated with DNA replication processes, which was also detected as enriched in the GO analyses of individual time points. A set of 48 genes (out of the 1781 genes) were affected in common at all three time points, but not with a consistent direction of expression change at all time points (Fig C in S1 Text), thus not being included in the GO enrichment analysis. The vast majority of the 1781 genes exhibited a sustained gene expression change all along the HDACi treatment period, being either sustainably up- or down-regulated across the three time points (12, 24 and 48 h) (Fig C in S1 Text). S3 Table gives the list of genes in each enriched GO category present in this analysis.

**Table 5:** Gene ontology categories enrichment for sustained differentially expressed genes detected as up-regulated or down-regulated in schistosomula, which were consistently affected across all the three time points of treatment with HDAC inhibitor (TSA).

Gene Ontology Category	GO ID	NSP	*p*-value^a^	Pop.	DEGs
**Genes sustainably up-regulated (820) at all time points of treatment with TSA**					
DNA metabolic process	GO:0006259	B	0.000493	132	29
Purine nucleotide binding	GO:0017076	M	0.009014	728	88
DNA replication	GO:0006260	B	0.009014	54	16
ATP binding	GO:0005524	M	0.025829	589	83
Cell projection part	GO:0044463	C	0.028029	6	4
**Genes sustainably down-regulated (913) at all time points of treatment with TSA**					
Receptor activity	GO:0004872	M	1.17E-05	118	35
Cell surface receptor signaling pathway	GO:0007166	B	0.000109	136	36
Membrane	Go:0016020	C	0.000109	583	105
Structural constituent of cytoskeleton	GO:0005200	M	0.002928	12	6
Integral component of membrane	GO:0016021	C	0.004328	586	115
Molecular transducer activity	GO:0060089	M	0.008249	140	31
Proteolysis	GO:0006508	B	0.008249	205	35
GTP binding	GO:0005525	M	0.027931	136	21

NSP, the namespace, or subontology; B, Biological process; C, Cellular component; M, Molecular function; Pop., population number of *S*. *mansoni* genes present in the GO category; DEGs, number of differentially expressed genes enriched in the category.

^a^Benjamini-Hochberg adjusted *p*-value.

**Fig 4:**
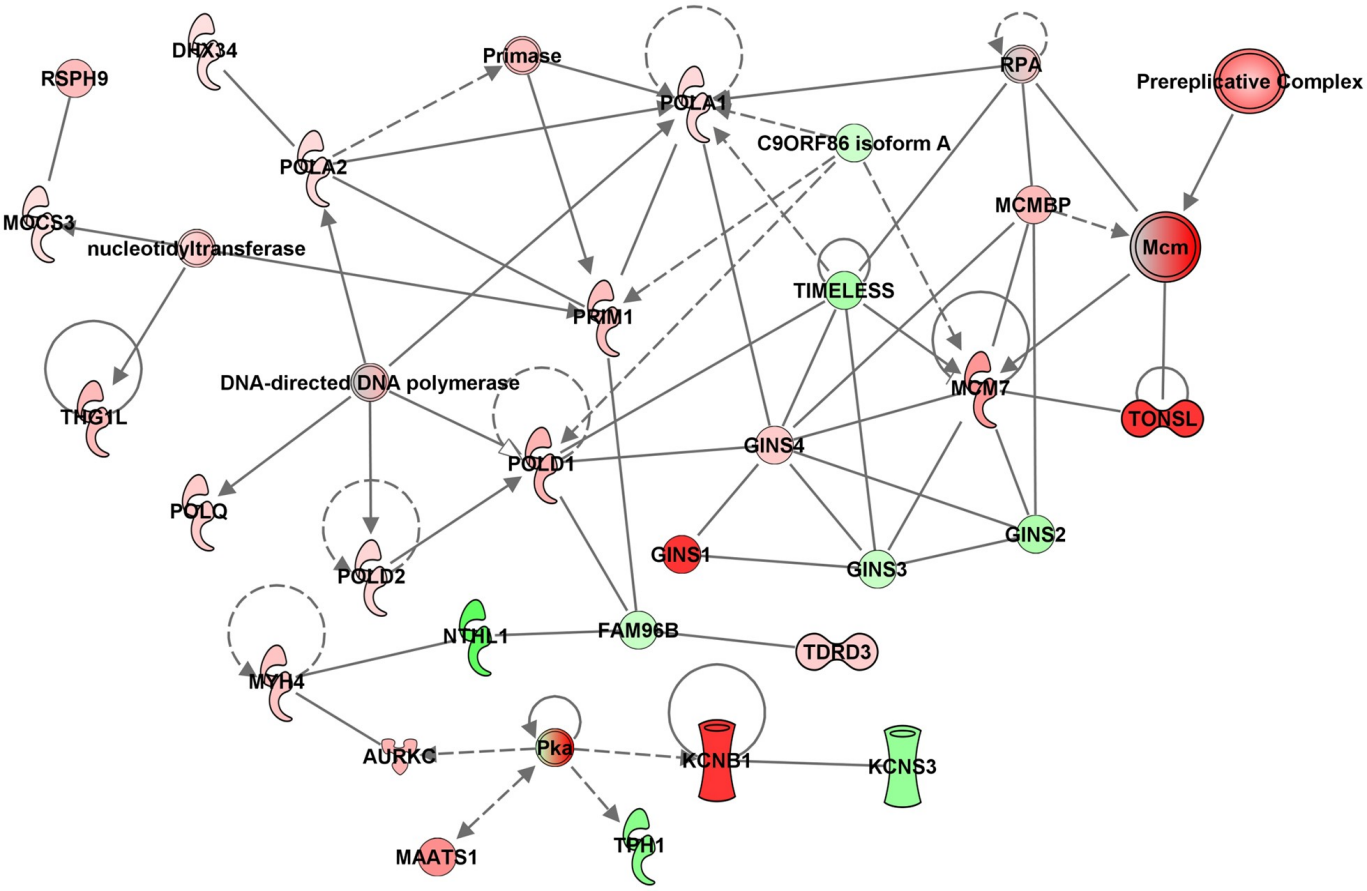
Enriched gene network involving DNA replication, recombination and repair affected by HDACi treatment of schistosomula. Ingenuity Pathway Analysis (IPA) identified of a significantly enriched gene network of differentially expressed genes, following a 24 h treatment of schistosomula with TSA. The network is associated with DNA replication, recombination and repair, and includes relevant genes such as those encoding the minichromosome maintenance (MCM) complex, Polymerases and Prereplication complex organization (ORC proteins). Up-regulated genes affected by TSA are colored in red and down-regulated genes are colored in green; shading intensity is according to the Log_2_ ratio (treated/control) value. Solid lines indicate direct interactions and dashed lines indicate indirect interactions between molecules. S4 Table gives the corresponding Smp gene information for each gene symbol present in this figure.

Consistent with the GO analysis, Ingenuity Pathway Analysis (IPA) also pointed to an enriched network of genes from the DNA replication mechanism, with most of them detected as up-regulated by the HDACi treatment after 24 h (Fig 4). It is interesting to note the presence of a set of genes encoding DNA polymerases, pre-replication complex organization, GINS complex and minichromosome maintenance (MCM) proteins; all these proteins are closely involved in the initiation, regulation and progression of DNA replication.

**Fig 5:**
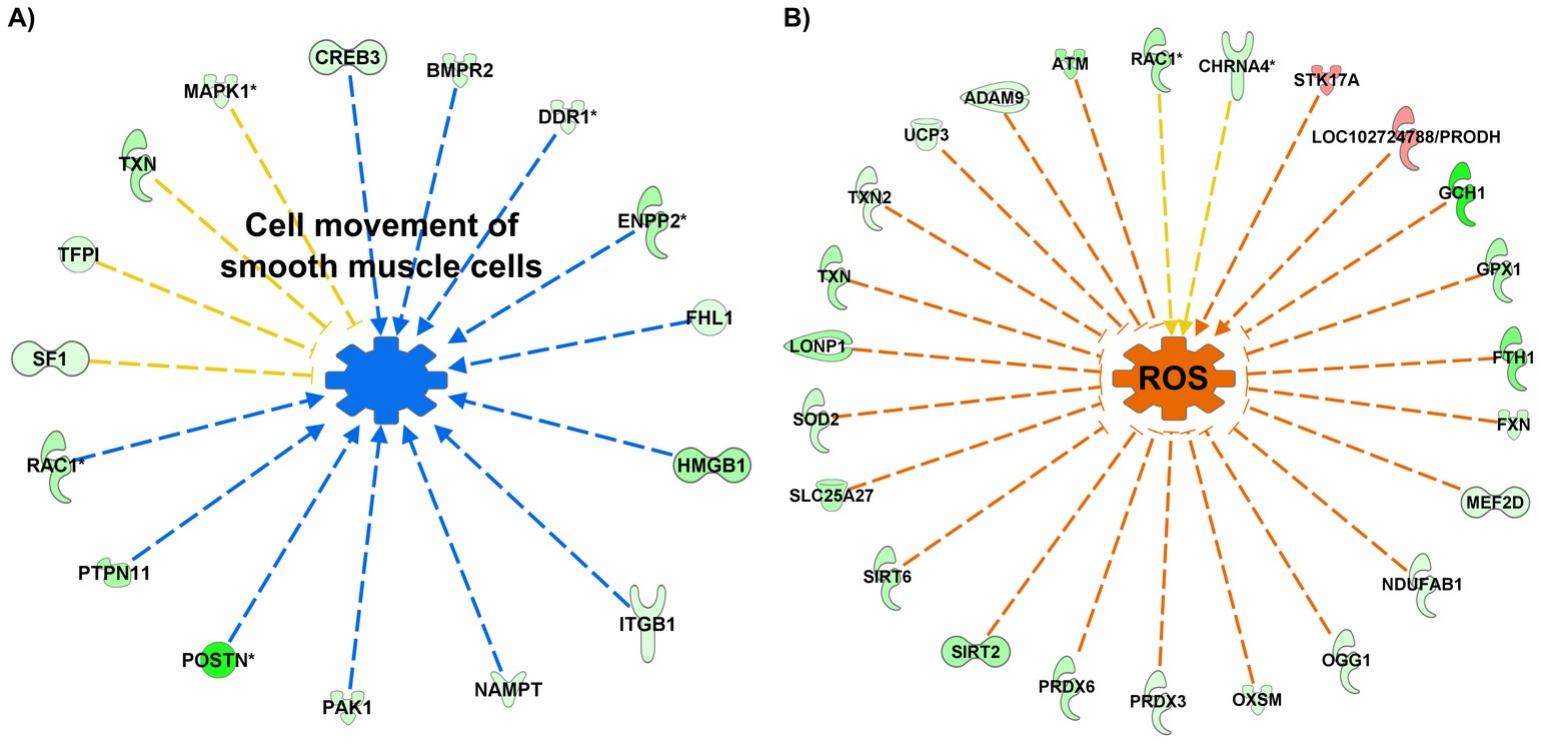
Cellular functions misregulated by HDACi after 48 h schistosomula treatment. IPA analysis pointed to the “cell movement of smooth muscle cells” cellular function (A) that could be functionally limited due to the down-regulated transcription of all involved genes. (B) The cellular function “control of the quantity of reactive oxygen species (ROS)” may be decreased in TSA-treated parasites due to the fact that 20 out of 22 genes that encode proteins responsible for reducing free radical generation are down-regulated in schistosomula upon TSA treatment, and 2 out of 4 genes that increase ROS are up-regulated. Genes down-regulated by the HDACi treatment are colored in green and genes up-regulated are colored in red. Arrows indicate the predicted effect downstream of the gene according to the gene expression pattern; blue arrow shows a predicted inhibition of the function indicated at the center; orange arrow shows a predicted increase of the cellular function indicated at the center, and yellow arrows show inconsistent prediction of the function according to the gene expression pattern. S5 Table gives the corresponding Smp gene information for each gene symbol present in this figure.

Upon longer exposure to HDACi (48 h treatment), two different enriched gene networks were detected, with most of the genes being down regulated, and being involved in cell movement of smooth muscle cells (Fig 5A) and in the production of reactive oxygen species (Fig 5B).

### Validation by quantitative PCR of genes detected by microarrays as affected by HDACi

**Fig 6:**
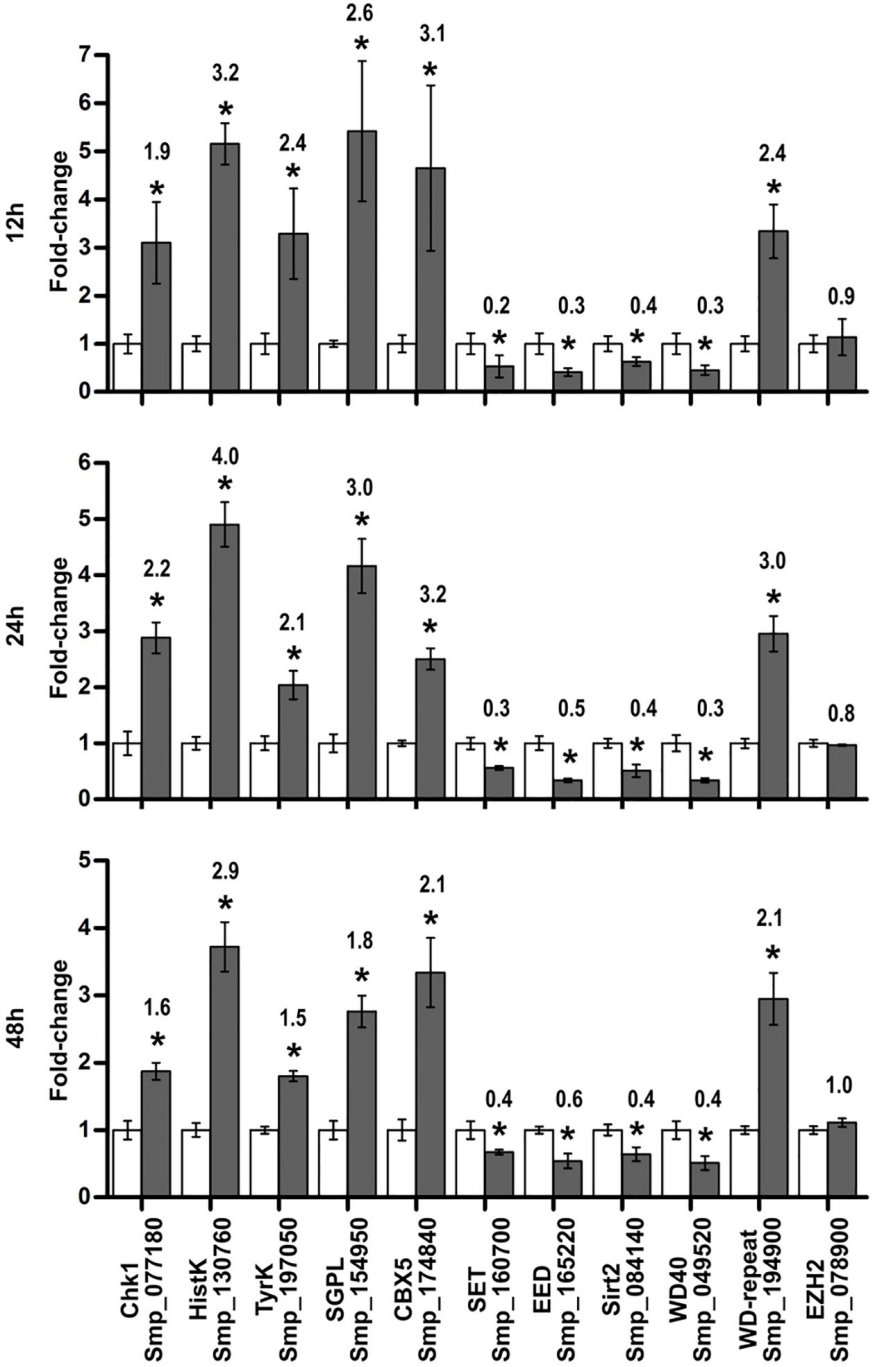
Validation by RT-qPCR of differentially expressed genes detected by microarray. Eleven selected genes with expression induced, repressed or not affected by treatment of schistosomula with 1 μM TSA for 12, 24 and 48 h were analyzed by relative quantitative PCR method normalized by the housekeeping PSMD gene (Smp_000740). We compared TSA treated samples (solid bars) with controls (open bars), and the graphs show the mean ± S.D. of three biological replicates for each condition. Statistical significance was evaluated with the t-test and significant changes are marked with asterisk (*p*-value ≤ 0.05). For comparison, we report above each solid bar the mean fold-change measured by microarray.

A set of differentially expressed genes was selected for RT-qPCR validation of the microarray results (six up-regulated genes, four down-regulated genes); selection was based on the following criteria: genes involved in signaling pathways such as *Sm*Chk1, *Sm*HistK, *Sm*TyrK and *Sm*SGPL, and also genes that encode proteins participating in chromatin remodeling such as *Sm*CBX5, *Sm*EED, *Sm*SET, *Sm*Sirt2, *Sm*WD40 and *Sm*WD-repeat. A statistically significant change of expression was detected by RT-qPCR for all selected genes at all three time-points of TSA treatment under analysis (Fig 6). The same fold-change pattern was detected both by qPCR and microarray, corresponding to a Pearson correlation greater than 0.95 for 12, 24 and 48 h. As a control, *Sm*EZH2 histone methyl-transferase, not differentially expressed in the microarray, was also included in the RT-qPCR (Fig 6, rightmost bars).

### Histone H3 acetylation in promoter regions regulates gene expression in parasites under the effect of HDACi

The overall increased acetylation of histones and the genome-wide gene expression regulation that were observed, led us to investigate the possible increased occupation by acetylated histone of the promoter region upstream of genes that were detected as up-regulated upon HDACi exposure. For this, we performed chromatin immunoprecipitation (ChIP) with antibodies against the histone marks related to transcription activation, namely H3K9ac, H3K14ac and H3K4me3 followed by qPCR using primers targeting the specific genomic DNA sequences of promoter regions of a set of selected genes that were up-regulated by TSA after 12 h treatment.

**Fig 7:**
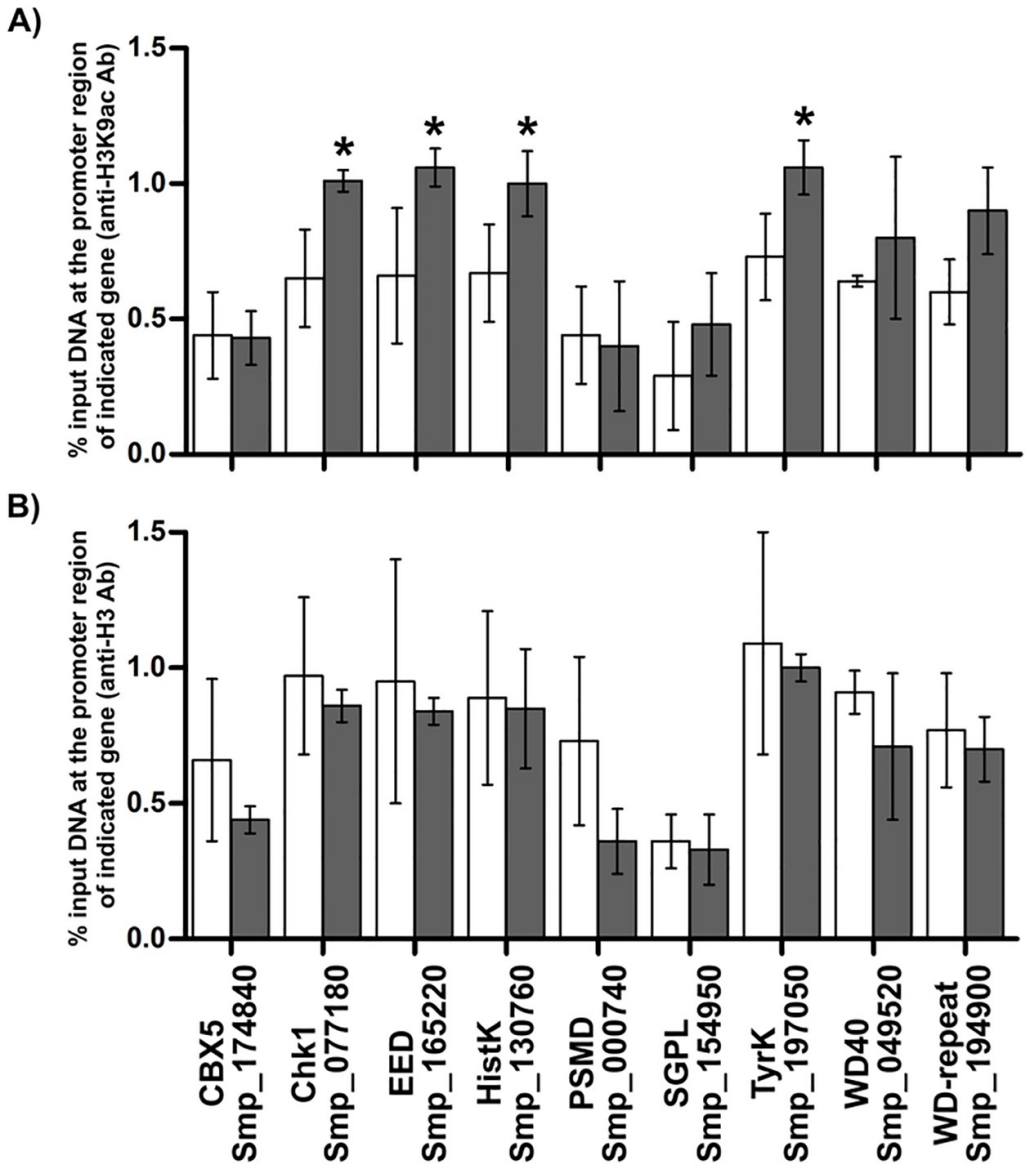
ChIP-qPCR targeting H3K9ac and total H3 at promoter region of differentially expressed genes. Chromatin immunoprecipitation (ChIP) was performed with antibodies anti-H3K9ac (A) or anti-H3 (B) and DNA from schistosomula treated for 12 h with 1 μM TSA (solid bars) or with vehicle (open bars). DNA from the promoter regions of the indicated selected genes that was present in the immunoprecipitated fraction was quantified by qPCR with specific primers. Results are presented as % input DNA at the indicated target promoter region normalized by % input DNA at the promoter of the non-expressed reference gene *Sm*Val19 (Smp_123090), as described in the Methods. Three independent biological replicates were analyzed. Statistical significance of enrichment was evaluated using t-test; asterisk indicates *p*-value ≤ 0.05.

We detected a significant increase of the H3K9ac mark at the promoter region of four out of eight chosen genes in schistosomula treated with TSA (Fig 7A); this result is corroborated by the fact that the total histone H3 occupancy at the promoter regions for all eight tested genes is not affected by TSA treatment (Fig 7B). H3K14ac did not show an increased occupancy at any of the promoter regions tested (Fig E in S1 Text). Also, the unrelated transcription repression histone mark H3K27me3 (Fig E in S1 Text) was not found enriched in any of the tested genes as expected, corroborating with western blotting assay where no change in this histone mark was detected.

### A number of genes encoding proteins with histone reader motifs were differentially expressed

Using the Blastp tool we searched for *S*. *mansoni* genes encoding proteins with histone reader motifs, and we identified 195 histone readers among the parasite expressed genes. Interestingly, many of them were detected as differentially expressed (q-value ≤ 0.05) after 12 h treatment (73 genes), 24 h (89 genes) and 48 h treatment (85 genes) (Table 1), although the number of affected genes was not sufficient to cause the class of histone readers to be significantly enriched. Most of these differentially expressed histone reader genes have the histone reader domain recognizing histone methyl-lysine, such as Ankyrin, WD40 and PHD domains (S2 Table). These genes encode key proteins in the regulation of chromatin remodeling complexes recruiting proteins with the ability of writing or erasing histone modifications.

Notably, among the differentially down-regulated genes we found SmEED (Smp_165220), which encodes a component of the Polycomb Repressor Complex 2 (PRC2) and contains 7 repeat-units of WD40 motifs that are necessary for EED to recognize histone H3K27me3 [49]. EED is responsible for the regulation of EZH2 methyl-transferase activity of PRC2, which inserts the H3K27me3 histone mark that determines transcription inhibition [49].

### Synergic effect of HDAC and EZH2 inhibition in schistosomula increases the parasite mortality

Having found that SmEED, an activator of EZH2, was down-regulated in the presence of TSA, we hypothesized that a small molecule inhibitor of SmEZH2 methyltransferase might increase parasite mortality when given simultaneously with TSA. To evaluate SmEZH2 as a possible new anti-parasite epigenetic target, we tested GSK343, a compound identified in human cancer cells as a highly potent, selective EZH2 inhibitor [21]. We assayed the *in vitro* effect of GSK343 on the viability of schistosomula after 4 days exposure and found that LD50 was 24.5 μM (Fig F in S1 Text).

**Fig 8:**
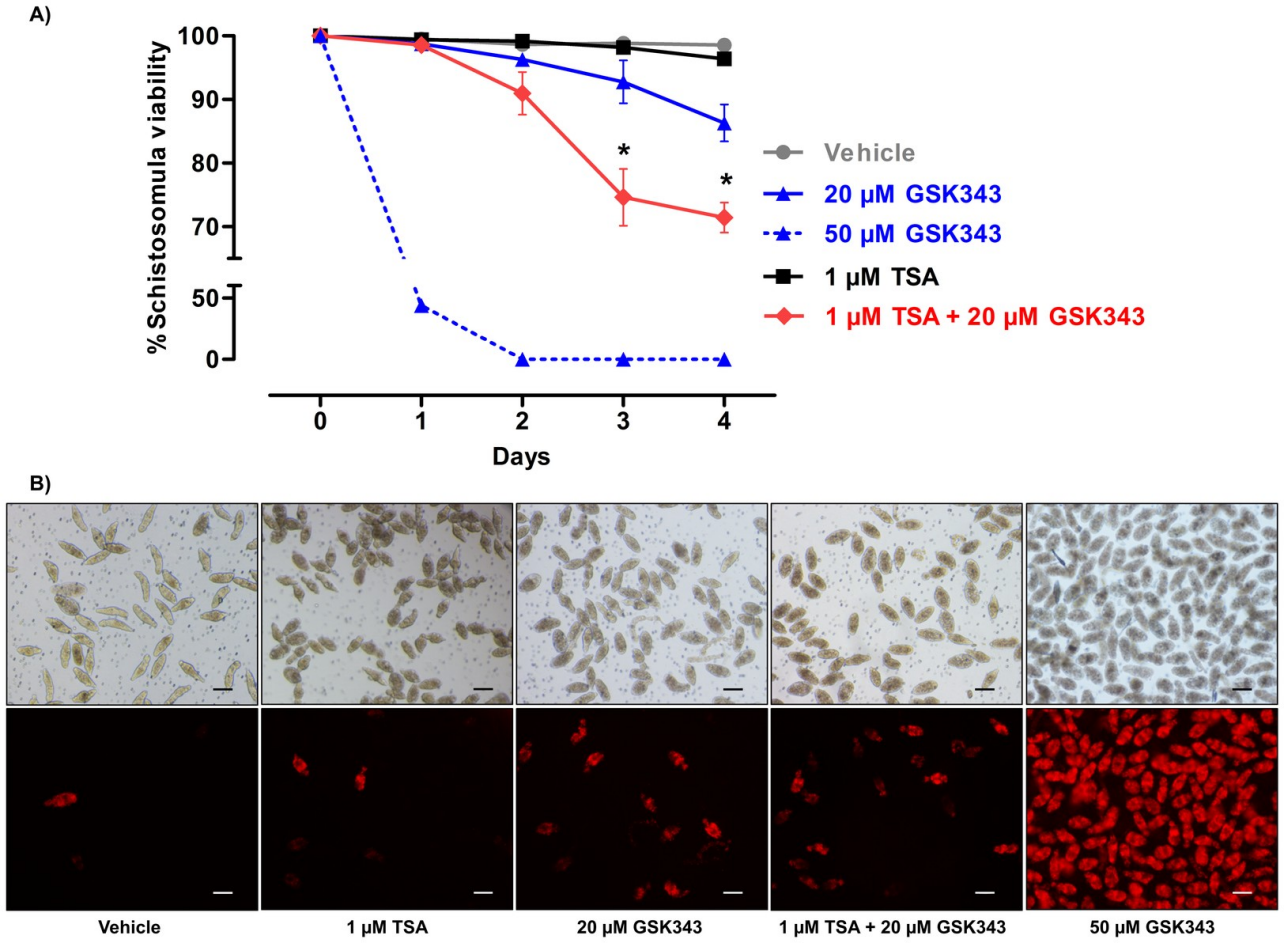
Synergistic effect of HDACi and EZH2i on schistosomula viability. (A) Schistosomula were treated with drugs in five different conditions: control with vehicle (DMSO + Ethanol); 20 μM GSK343; 50 μM GSK343; 1 μM TSA and 1 μM TSA + 20 μM GSK343. Parasites were observed in the microscope for four days, and photographed with light and fluorescence microscopy. The number of dead parasites (stained in red with propidium iodide) was counted and compared with the total number of parasites (counted in the light microscope). The percentages of live viable schistosomula were calculated along the four days, with a different pool of parasites being stained and analyzed independently at each day. The experiment was performed with six biological replicates, and the mean +/- SD is shown; for each biological replicate, two technical replicates were obtained, and at least 100 schistosomula were counted for each technical replicate. Significant decrease in viability when comparing GSK343 alone and GSK343 plus TSA was assessed with a two-way ANOVA statistical test; asterisks indicate *p*-value *≤* 0.001. (B) Typical images at day 4 are shown. The calibration bar shows 100 μm.

Next, we followed schistosomula viability along 4 days of treatment with GSK343 (Fig 8) and found zero viability of parasites at 50 μM GSK343 already after two days of treatment (Fig 8). At 20 μM GSK343, a concentration below LD50, 85% of schistosomula remained viable on day 4 (Fig 8). To test for the possible synergistic effect of both histone modifying enzyme inhibitors, we first exposed the parasites to a low dose of 1 μM TSA alone, a drug concentration which has previously been shown to cause very low mortality of schistosomula [15]. Indeed, we found that essentially 100% of schistosomula remained viable at day 4 of treatment with 1 μM TSA (Fig 8). Simultaneous exposure of schistosomula to 1 μM TSA plus 20 μM GSK343 caused a significant decrease in schistosomula viability to 70% after four days treatment, compared with 85% viability of schistosomula with 20 μM GSK343 alone (*p*-value ≤ 0.001 two-way ANOVA test) (Fig 8). The enhanced mortality of schistosomula caused by GSK343 in the presence of TSA compared with GSK343 alone is a clear indication of the synergistic effect of the two drugs.

### Homology modeling of a new parasitic epigenetic target

**Fig 9:**
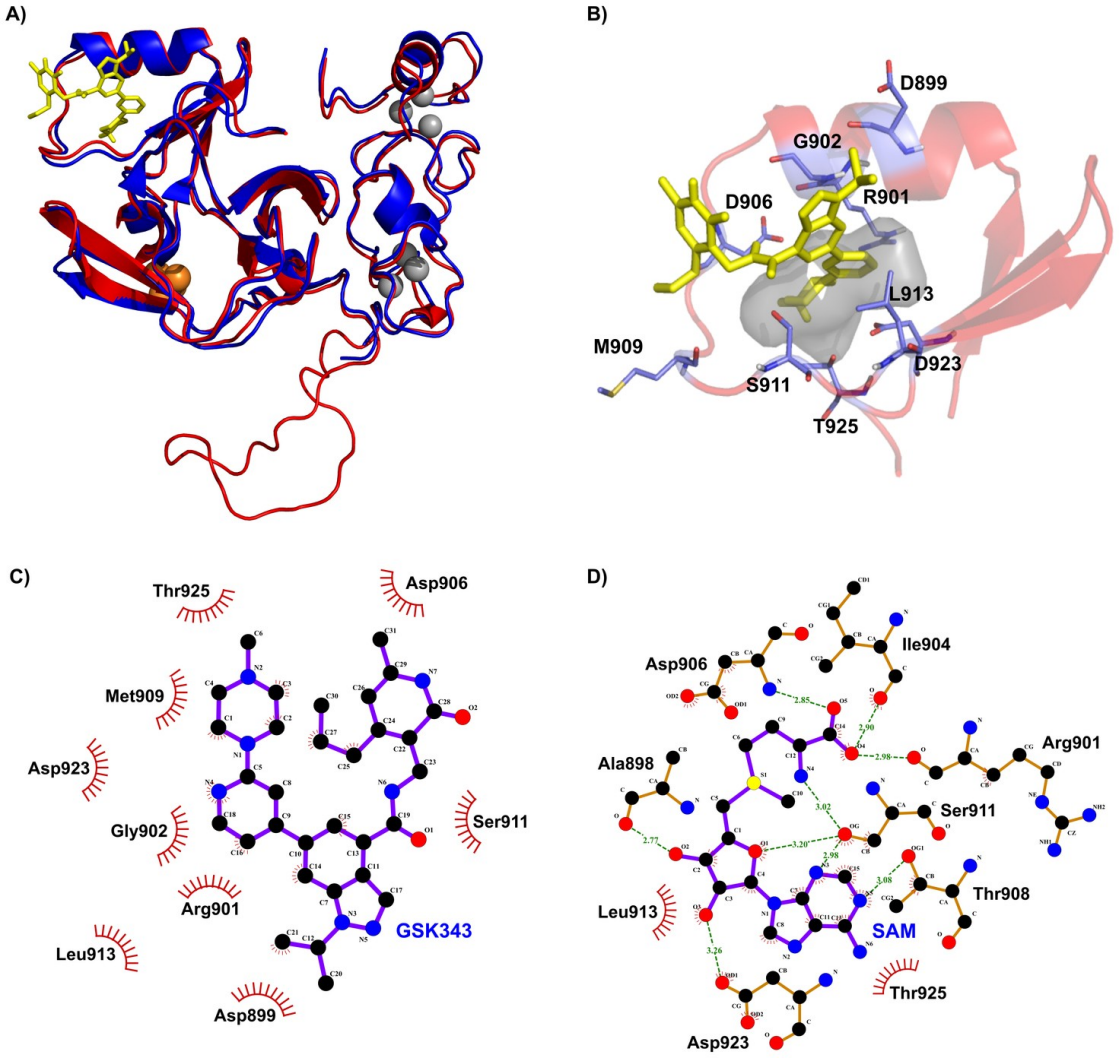
Docking pose of GSK343 in SmEZH2 and specific structural interactions of ligands. (A) Homology model of the SET domain of SmEZH2 colored red superimposed with hEHZ2 template colored blue, with docking pose of GSK343 (yellow) at the secondary pocket of SmEZH2. The orange spheres represent SO_4_ groups and grey spheres represent zinc ions. (B) Blown-up image of the secondary pocket of SmEZH2. Two-dimensional schematic overview of structural interactions observed in the SmEZH2 secondary pocket with (C) GSK343 or (D) cofactor SAM are shown at distances lower than 3.5 Å, with hydrophobic contacts represented by a red arc with spokes radiating towards the ligand atoms they contact and hydrogen bonds indicated by green dashed lines between the atoms involved.

Noting that GSK343 appears as an interesting compound with a schistosomicidal effect, we performed the homology modeling of the potential drug target SmEZH2 and computed the predicted binding energy between the compound and the enzyme. As a template we used hEZH2 which has 746 amino acids; just the methyltransferase SET domain (comprised of 233 amino acids) has the crystal structure solved [50]. The SmEZH2 gene (Smp_078900), in turn, encodes a protein with 1026 amino acids with a conserved SET domain, with 64% identity and 91% coverage to the hEZH2 SET domain. Alignment of the sequences comprising the SET domain from human EZH2, for which the structures with atomic resolution of 2 Å are available (4MI0 and 4MI5) with the sequence of *S*. *mansoni* EZH2 SET domain (Fig G in S1 Text) showed an identity higher than 60%, allowing the homology modeling of the SmEZH2 SET domain (Fig 9A and 9B). The obtained refined homology model evaluated by Molprobity [44] exhibited in Ramachandran plots 95.7% of its residues in favored regions and 99.1% in allowed regions with two outliers (Fig H in S1 Text), and ERRAT plots showed an overall quality factor of 93% for the SmEZH2 model structure. SmEZH2 presents an insertion of 19 amino acids at the SET domain compared to the hEZH2 (Fig G in S1 Text), which could not be modeled and possibly this fact has reduced the overall resolution of the achieved model; this insertion appears in the SmEZH2 model as a loop external to the region involved in catalysis (Fig 9A). In fact, SmHDAC8 insertions in the catalytic domain correspond to external solvent exposed loops that are not involved in catalysis as shown by X-ray crystallography [17] and the same is probably true of SmEZH2.

Using previous information of which amino acids interact with the cofactor SAM at the hEZH2 SET domain [46], we defined the region at SmEZH2 to be used in the predictions of docking of SAM to SmEZH2, that were computed with AutoDock Vina [47], also taking into consideration the competitive mode of inhibition of cofactor SAM and compound GSK343 in hEZH2, as shown in the literature [21]. This region is highly conserved between SmEZH2 and hEZH2 sequences, diverging only at V904I and Y908T. Compound GSK343 and SAM were predicted by the docking analyses to bind to the same protein region of SmEZH2 (Fig 9C and 9D), sharing common amino acids (Arg901, Asp906, Met909, Ser911, Leu913, Asp923 and Thr925) at a binding distance of 3.5 Å as indicated in Fig 9C and 9D, thus suggesting that GSK343 could act as a competitive inhibitor of SAM in SmEZH2.

The interaction between cofactor SAM and hEZH2 (Fig I in S1 Text) occurs at the same protein region as that predicted for SAM interaction with SmEZH2 (Fig 9D), and despite the conservation of sequence between hEZH2 and SmEZH2 in this region, just two amino acids predicted to be in close proximity to the SAM cofactor are in common, comparing SmEZH2 (Ser911 and Leu913, Fig 9D) and hEZH2 (Ser669 and Leu671, Fig I in S1 Text). The predicted binding energies of cofactor SAM to hEZH2 and SmEZH2 were similar (-6.3 ± 0.05 and -6.8 ± 0.27 kcal/mol). Notably, the predicted binding energies of inhibitor GSK343 to the models were more negative compared to the cofactor; GSK343 had a predicted binding energy of -7.83 ± 0.09 kcal/mol with SmEZH2, and for hEZH2 the binding energy was -8.1 ± 0.22 kcal/mol.

## Discussion

**Fig 10:**
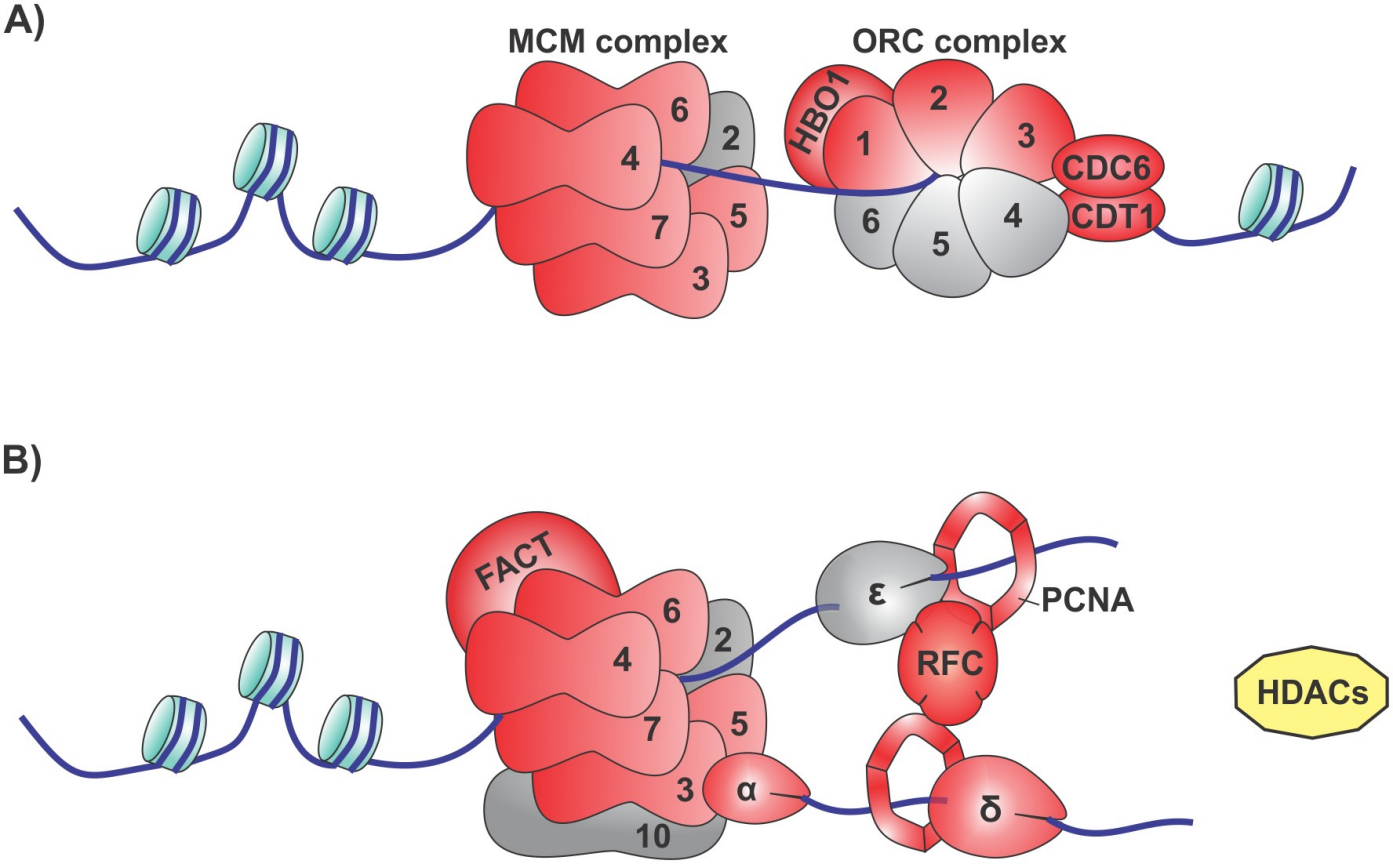
Schematic model of DNA replication machinery with *S*. *mansoni* genes affected by HDACi treatment. (A) Assembly of the pre-replication complex at the origin of DNA replication. (B) Eukaryotic replisome. TSA has affected the expression of genes encoding proteins belonging to the DNA replication machinery; here we show a representation of all genes involved in this process, and we indicate those with induced expression (red color) as well as those not affected (grey color). HDACs (yellow) are required for the maturation of chromatin, allowing the nucleosomes to assemble, whereas this process may be unsettled with the inhibition by TSA. S6 Table gives the corresponding Smp gene information for each gene symbol present in this figure.

We explored the effect of HDACi TSA on schistosomula gene expression, showing a large number of affected genes specifically related to different cellular functions. Remarkably, genes encoding proteins with activity at the DNA replication fork were up regulated, mainly after 24 h treatment, such as the genes responsible for Replication Complex organization (ORC1, ORC2, ORC3, CDC6, CDT1, MCM3, MCM4, MCM5, MCM6, MCM7, HBO1, FACT and RFC1-5) (Fig 10A). Acetylation of histones is closely associated with DNA replication, stimulating the replication activity at the origin, which is recognized by the origin recognition complex (ORC)–heterohexamer with DNA-dependent ATPase activity [51]. After ORC binds to the origin, factors CDC6 (cell division cycle 6) and CDT1 (DNA replication factor) are recruited and facilitate the loading of the MCM complex (minichromosome maintenance protein complex MCM2-7) with helicase activity, forming a ring around the replication origin, encircling the pre-replication complex (Pre-RC) [52]. CDT1 recruits a histone acetyltransferase (HBO1) to Pre-RC, which preferentially targets the histone H4 residues K5, K8 and K12, enhancing MCM2-7 loading through a mechanism requiring its acetyltransferase activity [53]. Further to this known mechanism involving a HAT, the replication fork also commits the histone chaperone FACT (facilitates chromatin transcription) that interacts with histones H2A-H2B and H3-H4 dimers promoting nucleosome disassembly and assembly [54]. We suggest that all this balanced mechanism of chromatin replication is being activated by HDAC inhibition, due to the increased gene expression of all the genes involved in the replication machinery. Also, the increased expression of genes encoding proteins from the replisome component, responsible for the replication initiation origin, fork progression and histone dynamics, as well the hyperacetylation of histones, suggests a genome-wide open chromatin status in the parasite due to the treatment with HDACi.

The mechanism of chromatin deacetylation is important for the maturation of nascent chromatin, and is required for fork progression and stability. In line with this, disruption of HDAC functions by HDACi directly affects replication and generates a reduction in the rate of replication fork progression [55]. We observed important genes with increased expression related to this process such as replication factor c (RFC1-5) and its downstream partner, PCNA (Fig 10B). As a central fork component, the heterotrimeric clamp PCNA, besides orchestrating DNA synthesis and stimulating DNA polymerases activity and nucleosome assembly, also recruits HDACs to chromatin maturation, as well as other maturation factors [52]. The up-regulation of PCNA, Pol alpha, and Pol delta with TSA treatment possibly affects the cohesin rings upon fork passage. We suggest that the increased expression of components of the replication machinery may cause a replication stress as a result of HDAC inhibition, creating the possibility for DNA damage, a process that has been shown to occur upon hyperacetylation of histones [55].

An important cellular function, namely control of the quantity of reactive oxygen species (ROS) appears to be decreased by TSA HDACi, because 20 out of 22 genes that encode proteins responsible for reducing free radicals generation are down-regulated in schistosomula upon TSA treatment, and 2 out of 4 genes that increase ROS are up-regulated. In fact, in human cancer cells HDACi are thought to cause apoptosis through the induction of DNA damage and genomic instability as a result of the generation of ROS [56].

Recently, it was shown that depletion of *Sm*CBP1 (Smp_105910), an HAT, resulted in an increase of neoblast proliferation [57], and in our data this gene is down regulated after 12 and 48 h treatment. In line with this finding, neoblast genes such as FGF receptor gene (Smp_175590) and Ago2 (Smp_179320) [58] are up-regulated after 24 h treatment, so we suggest that under the stress of a sub-lethal dose of TSA the parasite is promoting the proliferation of its somatic stem cells. In fact, stem cell proliferation for tissue regeneration is induced by apoptosis after tissue injury [59], and HDAC activity is an essential regulator of tissue regeneration in model organisms under the effect of HDACi [60,61].

We found that up to about half of the gene loci in *S*. *mansoni* showed antisense transcription and that 33 to 45% of total expressed antisense RNAs were differentially expressed upon TSA treatment (Table 1). Recently, a report on *S*. *japonicum* genes differentially expressed between genders has shown that a total of 685 and 430 genes were detected in males and females, respectively, as having significant fold-change values ≥ 2 simultaneously in the forward and the reverse strand [62]. Our results confirm that also in *S*. *mansoni*, a large number of genes exhibit antisense transcription, and that frequently the antisense transcripts show significant differential expression. Further experiments are needed to understand the role of these antisense messages in the parasite biology.

The epigenome of *S*. *mansoni* has been recently explored, associating the gene expression levels with chromatin modifications [30,37], or identifying chromatin epigenetic marks at the transcript start sites (TSSs) of genes [33]. Here we observed that on average the genes with a significantly enriched H3K4me3 mark at their TSSs showed an activation of transcription upon HDAC inhibition, whereas those genes without the mark showed on average an inhibition of transcription in the presence of TSA. In humans, it has already been described that the presence of the H3K4me3 mark at a gene TSS is often coupled with histone acetylation marks in the promoter region and gene body, allowing chromatin opening and transcription elongation [63]. Our results suggest that there is also a coupling between these two marks in *S*. *mansoni*. However, it should be noted that changes in transcription might not always be directly associated with hyperacetylation, for example when hyperacetylation affects the expression of a transcription factor (TF) that will in turn modify the expression of the TF target genes, regardless of whether the promoters of the latter are hyperacetylated or not.

TSA treatment affected the expression of dozens of histone reader genes. Histone readers are important components of the chromatin remodeling complexes, and are able to precisely recognize histone post-translational modifications and recruit components responsible for regulating transcription, DNA replication, DNA damage and chromatin remodeling [64]. Our observed change in expression of SmEED, a histone reader from the Polycomb Repressor Complex 2 (PRC2), caused by a sub-lethal dose of TSA, suggested that another component of PRC2, the EZH2 methyltransferase could be tested as target for inhibition. We therefore tested GSK343, an EZH2 inhibitor [21], and found that GSK343 was active *in vitro* against *S*. *mansoni* and acted synergistically with TSA, significantly increasing parasite death. This approach opens the perspective of using the information gathered here about the change in expression of the dozens of histone reader genes involved in regulation of the epigenetic program in *S*. *mansoni* as a starting point to look for possible novel schistosomicidal targets.

## Conclusions

We have shown that the TSA HDAC inhibitor, a known schistosomicidal drug, causes a wide range of gene expression changes in *S*. *mansoni*, and we were able to point to a number of cellular functions that were affected in the parasite, such as DNA replication and control of reactive oxygen species. A new epigenetic enzyme SmEZH2 emerged as a novel potential drug target to be studied with schistosomicidal activity, with its inhibition having a synergistic anti-parasitic effect along with HDAC inhibition.

## Supporting information

S1 Table List of primers used in qPCR and ChIP-qPCR. (XLSX)

S2 Table List of Smp genes and antisense RNAs with annotations and with expression data from microarray experiments at three different time points after exposure of *S*. *mansoni* schistosomula to the HDAC inhibitor TSA. (XLSX)

S3 Table Gene Ontology enrichment analyses—List of genes per GO category. (XLSX)

S4 Table Smp gene annotations for all genes shown in the enriched gene network involving DNA replication, recombination and repair affected by HDACi treatment of schistosomula (from Fig 4). (XLSX)

S5 Table Smp gene annotations for all differentially expressed genes from Fig 5. (XLSX)

S6 Table Smp gene annotations for all genes shown in the schematic model of the DNA replication machinery from Fig 10. (XLSX)

S1 Text This file contains additional information to this article, corresponding to: Table A. Summary information about transcript expression changes for both strands at three different time points after exposure of S. mansoni to HDAC inhibitor TSA; Fig A. Pearson correlation coefficients among replicates calculated with all expressed *S*. *mansoni* genes present in the microarray; Fig B. Venn diagrams with the number of up-regulated and down-regulated genes; Fig C. Gene expression profile of 1781 genes affected in common at the three analyzed time points in schistosomula treated with HDACi; Fig D. Cumulative distribution function of expression fold-changes for all differentially expressed genes separated per the presence or absence of histone mark H3K4me3; Fig E. ChIP-qPCR targeting the H3K14ac and H3K27me3 histone marks at the promoter regions of differentially expressed genes; Fig F. Median lethal dose LD50 for the EZH2 inhibitor GSK343 in schistosomula; Fig G. Alignment of amino acid sequences from the SmEZH2 SET domain and from two models of hEZH2; Fig H. Ramachandran plots of SmEZH2 3D model; Fig I. Two-dimensional schematic of specific structural amino acids of hEZH2 models with interactions and ligands.
(PDF)
