# Supplementary material for: Histone deacetylase inhibition modulates histone acetylation at gene promoter regions and affects genome-wide gene transcription in Schistosoma mansoni
Source: PLoS Negl Trop Dis. 2017 Apr 13;11(4):e0005539. doi: 10.1371/journal.pntd.0005539 (PMC5404884; doi:10.1371/journal.pntd.0005539)
Supplement: S1 Text — Summary information about transcript expression changes for both strands at three different time points after exposure of S. mansoni to HDAC inhibitor TSA; Fig A. Pearson correlation coefficients among replicates calculated with all expressed S. mansoni genes present in the microarray; Fig B. Venn diagrams with the number of up-regulated and down-regulated genes; Fig C. Gene expression profile of 1781 genes affected in common at the three analyzed time points in schistosomula treated with HDACi; Fig D. Cumulative distribution function of expression fold-changes for all differentially expressed genes separated per the presence or absence of histone mark H3K4me3; Fig E. ChIP-qPCR targeting the H3K14ac and H3K27me3 histone marks at the promoter regions of differentially expressed genes; Fig F. Median lethal dose LD50 for the EZH2 inhibitor GSK343 in schistosomula; Fig G. Alignment of amino acid sequences from the SmEZH2 SET domain and from two models of hEZH2; Fig H. Ramachandran plots of SmEZH2 3D model; Fig I. Two-dimensional schematic of specific structural amino acids of hEZH2 models with interactions and ligands. (PDF) [file pntd.0005539.s007.pdf]

Supporting Information for

Histone deacetylase inhibition modulates histone acetylation at gene promoter regions and affects genome-wide gene transcription in *Schistosoma mansoni*

Letícia Anderson[1,2]; Monete Rajão Gomes[1,2]; Lucas Ferreira daSilva[1,2]; Adriana da Silva Andrade Pereira[1,2]; Marina M. Mourão[3]; Christophe Romier[4]; Raymond Pierce[5]; Sergio Verjovski-Almeida[1,2*]

1 – Departamento de Bioquímica, Instituto de Química, Universidade de São Paulo, 05508-900 São Paulo, SP, Brazil; 2 – Instituto Butantan, 05503-900 São Paulo, SP, Brazil. 3 – Grupo de Helmintologia e Malacologia Médica, Centro de Pesquisas René Rachou, Fundação Oswaldo Cruz, 30190-002 Belo Horizonte, MG, Brazil; 4 - Départment de Biologie Structurale Intégrative, Institut de Génétique et Biologie Moléculaire et Cellulaire (IGBMC), Université de Strasbourg, CNRS, INSERM, 1 rue Laurent Fries, 67404 Illkirch Cedex, France. 5 - Univ. Lille, CNRS, Inserm, CHU Lille, Institut Pasteur de Lille, U1019 UMR 8204CIIL - Centre d'Infection et d'Immunité de Lille, F-59000 Lille, France

*Corresponding author e-mail: verjo@iq.usp.br

Table A. Summary information about transcript expression changes for both strands at three different time points after exposure of *S. mansoni* to HDAC inhibitor TSA

mRNA and asRNA from the same locus	12 h	24 h	48 h
mRNA up-regulated and asRNA up-regulated	635	718	262
mRNA down-regulated and asRNA down-regulated	255	104	462
mRNA up-regulated and asRNA down-regulated	28	40	134
mRNA down-regulated and asRNA up-regulated	4	8	4
mRNA not differentially expressed and asRNA up-regulated	401	365	502
mRNA not differentially expressed and asRNA down-regulated	66	39	81

	12h_rep1	12h_rep2	12h_rep3	24h_rep1	24h_rep2	24h_rep3	48h_rep1	48h_rep2	48h_rep3
12h_rep1	1								
12h_rep2	0.8837	1							
12h_rep3	0.7472	0.8397	1						
24h_rep1	0.8817	0.8575	0.7747	1					
24h_rep2	0.8279	0.7627	0.6896	0.8616	1				
24h_rep3	0.8594	0.8584	0.7906	0.8739	0.8603	1			
48h_rep1	0.7052	0.7703	0.6602	0.8034	0.6875	0.7693	1		
48h_rep2	0.7943	0.7812	0.6642	0.8504	0.8415	0.8768	0.8405	1	
48h_rep3	0.7264	0.6920	0.5884	0.7866	0.7174	0.8170	0.8082	0.8721	1

Fig A. Pearson correlation coefficients among replicates calculated with all expressed *S. mansoni* genes present in the microarray.

Pearson correlation coefficients were calculated using the values of the Log_2 ratio (treated/vehicle) for each gene among the biological replicates (rep1 to rep3) and treatment time points (12h, 24h and 48h), as indicated in the headings of the columns and rows. The shades of red represent the different values along the range of calculated correlation coefficients.

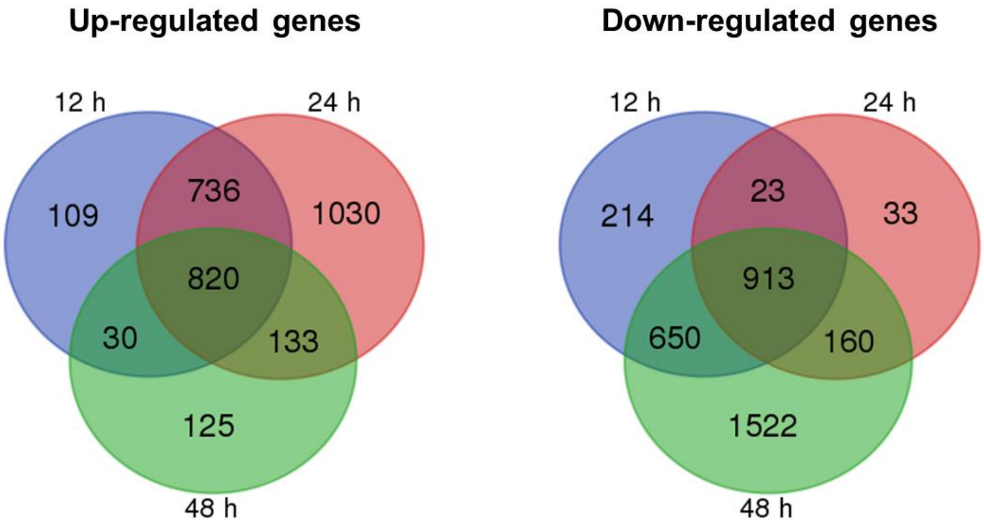

Fig B. Venn diagrams with the number of up-regulated and down-regulated genes. The panel at left shows the subset of genes that were up-regulated at the three time points after exposure of schistosomula to HDACi TSA (12, 24 and 48 h), whereas the right panel shows the subset of genes that were down-regulated.

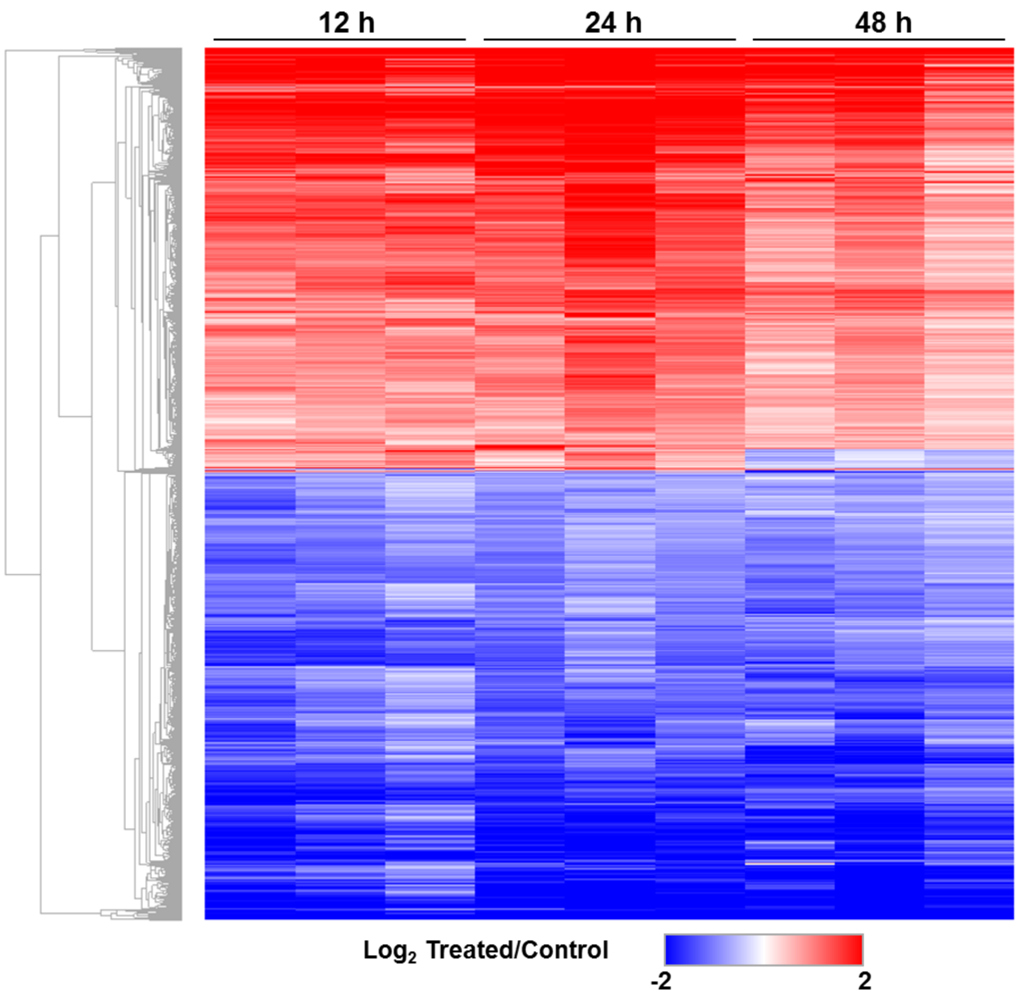

Fig C. Gene expression profile of 1781 genes affected in common at the three analyzed time points in schistosomula treated with HDACi.

Heatmap for a total of 1781 differentially expressed genes (each on one horizontal line) that were affected in common at the three different time points after exposure of schistosomula to HDACi TSA (12, 24 and 48 h), as indicated at the top of the heatmap. Three biological replicates (three adjacent columns) were obtained at each time point. Gene expression levels are calculated as Log_2 ratio (treated/vehicle) and color-coded as indicated on the scale at the bottom.

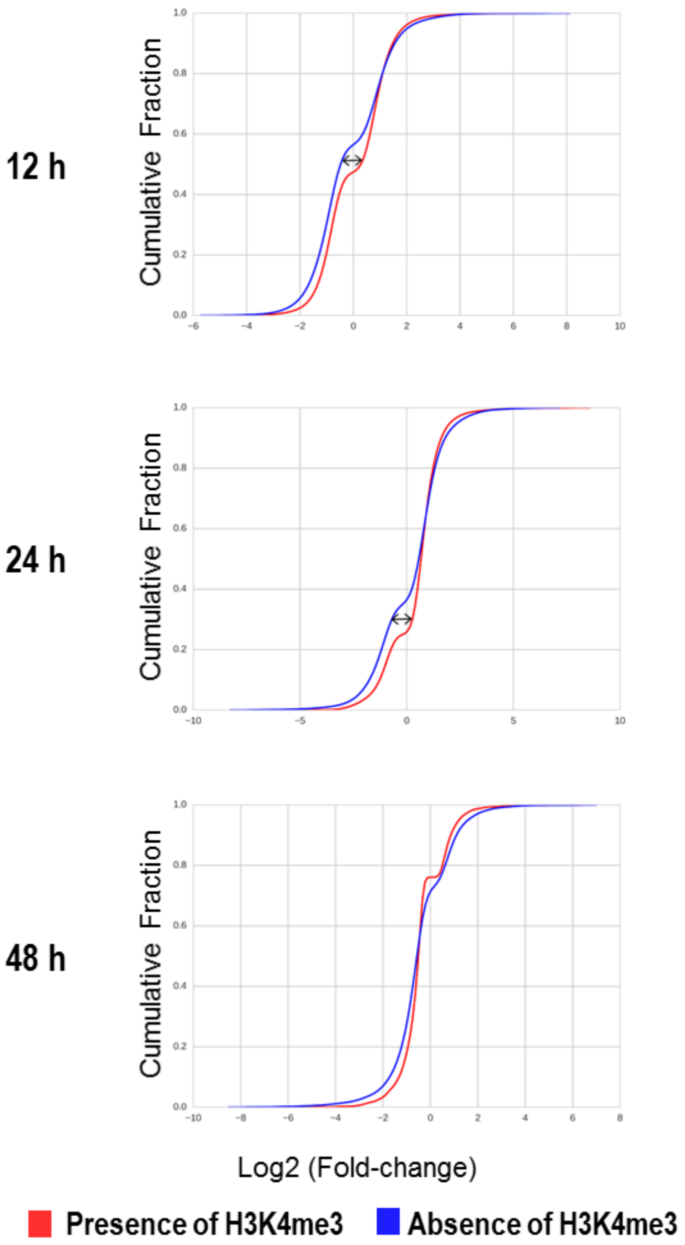

Fig D. Cumulative distribution function of expression fold-changes for all differentially expressed genes separated per the presence or absence of histone mark H3K4me3.

Gene expression fold-change (log2) of differentially expressed genes that were separated into two groups according to the presence (red line) or absence (blue line) of histone mark H3K4me3 at their TSSs. Internal black arrow points to the difference between the two analyzed groups, highlighting a shift towards the right in the cumulative distribution of gene expression changes at 12 and 24 h, for the genes with the presence of the H3K4me3 mark at their promoter regions, compared with genes without this histone mark.

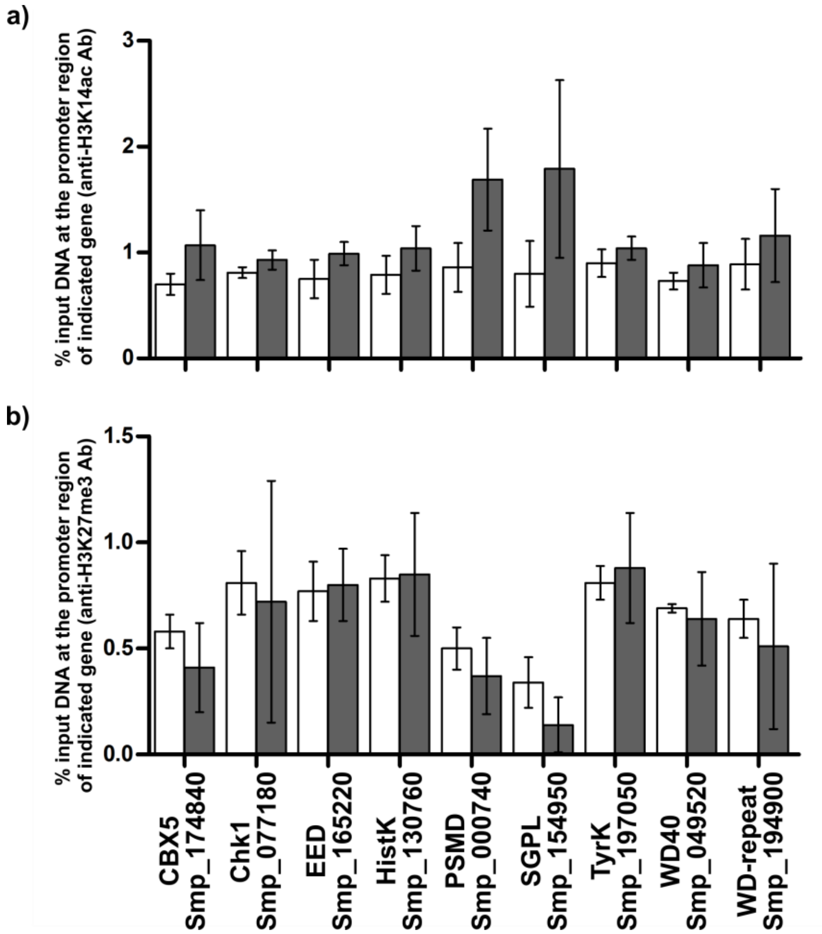

Fig E. ChIP-qPCR targeting the H3K14ac and H3K27me3 histone marks at the promoter regions of differentially expressed genes.

Chromatin immunoprecipitation (ChIP) was performed with antibodies anti-H3K14ac (panel a) or anti-H3K27me3 (panel b) in schistosomula treated for 12 h with 1 μM TSA (solid bars) or with vehicle (open bars) and the DNA from the promoter regions of the indicated selected genes that was co-immunoprecipitated was quantified by qPCR with specific primers. Results are presented as % input DNA at the indicated target promoter region normalized by % input DNA at the promoter of the non-expressed reference gene *Sm*Val19 (Smp_123090), as described in the Methods. Three independent biological replicates were analyzed. Statistical significance of enrichment was evaluated using t-test; asterisk indicates p-value ≤ 0.05.

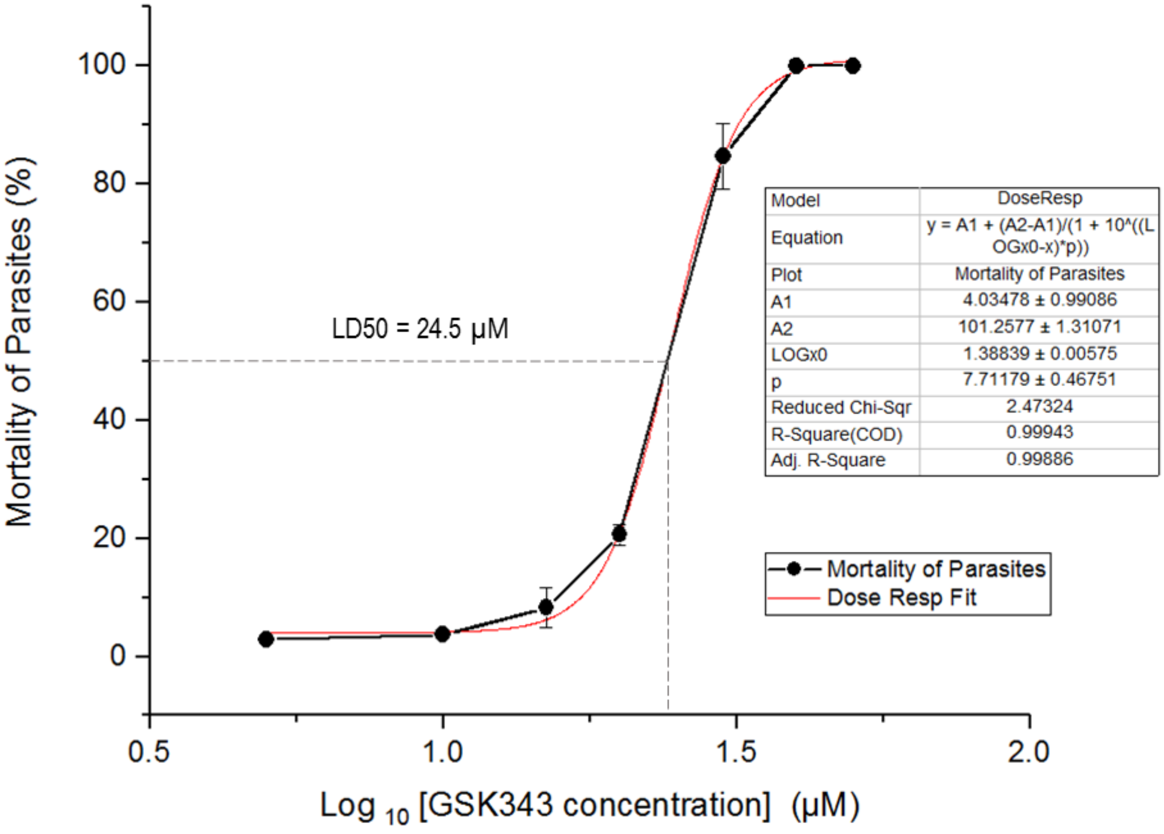

Fig F. Median lethal dose LD50 for the EZH2 inhibitor GSK343 in schistosomula.

Median lethal dose LD50 was determined as the concentration of GSK343 that caused 50 % of schistosomula parasites in *in vitro* culture to die after 96 hours treatment. LD50 was calculated using seven GSK343 doses (5 µM, 10 µM, 15 µM, 20 µM, 30 µM, 40 µM and 50 µM); for each concentration, over 200 parasites were observed in a Petri dish in the microscope with visible light and UV light, photographed and counted as alive or dead. Dead parasites were stained with propidium iodide and detected as red images under UV light. The percentage of dead parasites (mortality of parasites) was calculated from the images countings. Three biological replicates were obtained and the values (black circles) represent the mean ± S.D. A sigmoidal function has been fitted to the points (red curve) and an adjusted coefficient of determination (COD) adjusted R^2-value > 0.99 was calculated; the fitted LD50 = $10^{1.38839}$ = 24.5 µM.

```
SmEZH2   HHYHPCDHPGQRCDDSCSCRIAGTFCEKFCQCPPDCPNRFLGCRCRGQCNTKLCPCVLAV 805
4MI0     --YQPCDHPRQPCDSSCPCVIAQNFCEKFCQCSSECQNRFPGCRCKAQCNTKQCPCYLAV 58
4MI5     GSLQPCDHPRQPCDSSCPCVIAQNFCEKFCQCSSECQNRFPGCRCKAQCNTKQCPCYLAV 60
            :***** * **.** * ** .********  :* *** ****:.***** *** ***

SmEZH2   RECDPDLCLSCGAHSSFRSFASGNSMDLLSLLQTTLPPVTGTCRNVAIQRGWRKHLLMAP 865
4MI0     RECDPDLCLTCGAADHWDSK-------------------NVSCKNCSIQRGSKKHLLLAP 99
4MI5     RECDPDLCLTCGAADHWDSK-------------------NVSCKNCSIQRGSKKHLLLAP 101
         *********:*** . : *                    . :*:* :**** :****:**

SmEZH2   SDVAGWGIFIKEAAEKNDFIYEYCGEIISQDEADRRGKIYDKTMSSFLFNLNRDFVVDAT 925
4MI0     SDVAGWGIFIKDPVQKNEFISEYCGEIISQDEADRRGKVYDKYMCSFLFNLNNDFVVDAT 159
4MI5     SDVAGWGIFIKDPVQKNEFISEYCGEIISQDEADRRGKVYDKYMCSFLFNLNNDFVVDAT 161
         ***********: .:**:** *****************:*** *.*******.*******

SmEZH2   RKGNKIRFANHSVNPNCHAKVIMVNGDHRIGIFAKRAILPGEELFFDYRYGPT------- 978
4MI0     RKGNKIRFANHSVNPNCYAKVMMVNGDHRIGIFAKRAIQTGEELFFDYRYSQADALKYVG 219
4MI5     RKGNKIRFANHSVNPNCYAKVMMVNGDHRIGIFAKRAIQTGEELFFDYRYSQADALKYVG 221
         *****************:***:****************  **********. :

SmEZH2   -------------- 978
4MI0     IEREMEIPHHHHHH 233
4MI5     IEREMEIP------ 229
```

Fig G. Alignment of amino acid sequences from the SmEZH2 SET domain and from two models of hEZH2.

Multiple sequence alignment of SmEZH2 with hEZH2 models (4MI0 and 4MI5) used to perform the modeling. Amino acid numbers refer to the SmEZH2 full sequence.

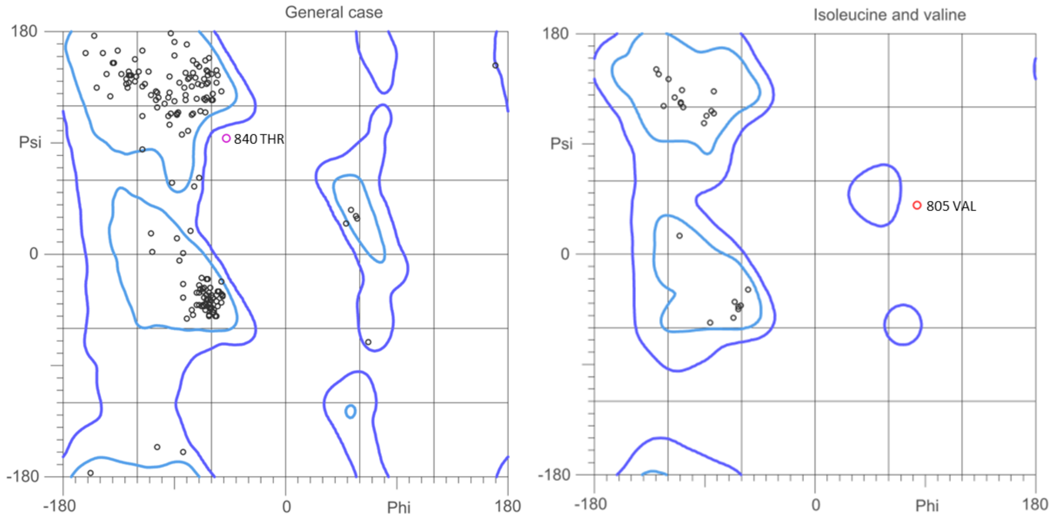

Fig H. Ramachandran plots of SmEZH2 3D model.

Ramachandran plot of amino acids from 746 - 978 of refined SmEZH2 SET domain model with 95.7% (221/233) of residues in favored regions indicated by light blue and 99.1% (229/233) of residues in allowed regions indicated by dark blue, as analyzed by Molprobity. There were 2 outliers (phi, psi): 840 THR (-48.7, 94.5) indicated in a pink circle at the general case plot and 805 VAL (83.5, 40.3) indicated in a red circle at the isoleucine and valine plot.

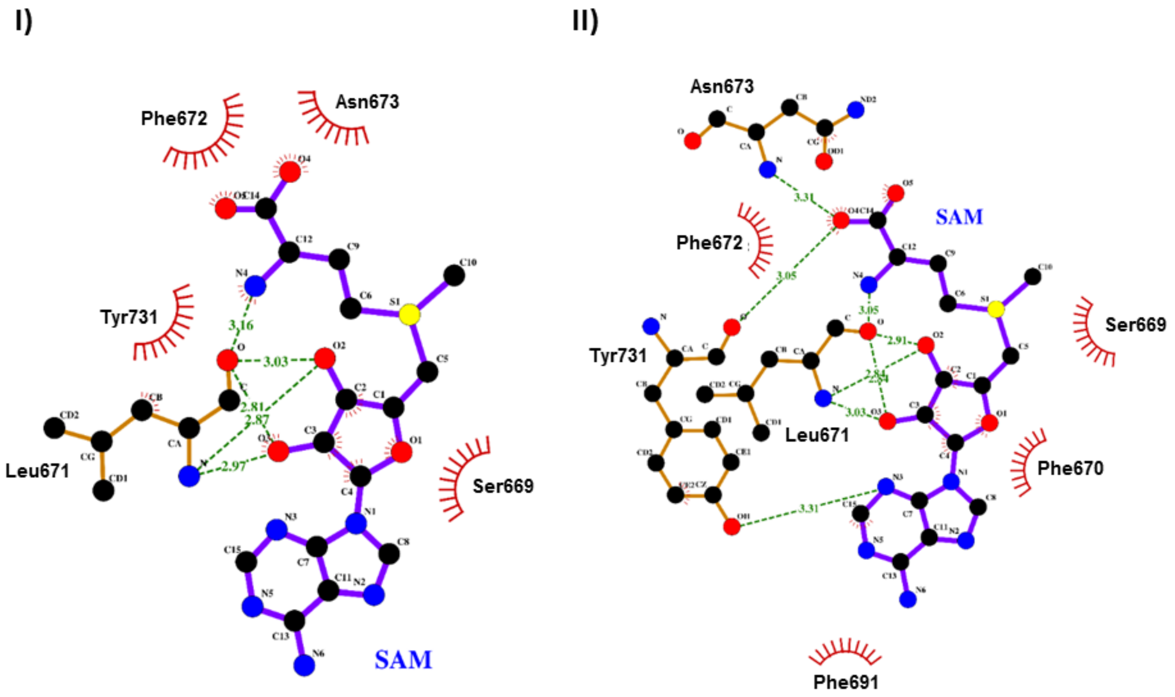

Fig I. Two-dimensional schematic of specific structural amino acids of hEZH2 models with interactions and ligands.

Two-dimensional schematic overview of structural interactions, showing amino acids that interact with cofactor SAM, taken from hEZH2 structural models 4MI0 (I) and 4MI5 (II). A red arc represents hydrophobic contacts with spokes radiating towards the ligand atoms they contact, and hydrogen bonds indicated by green dashed lines between the atoms involved.
